# Pneumococcal galactose catabolism is controlled by multiple regulators acting on pyruvate formate lyase

**DOI:** 10.1038/srep43587

**Published:** 2017-02-27

**Authors:** Firas A. Y. Al-Bayati, Hasan F. H. Kahya, Andreas Damianou, Sulman Shafeeq, Oscar P. Kuipers, Peter W. Andrew, Hasan Yesilkaya

**Affiliations:** 1 Department of Infection, Immunity & Inflammation, University of Leicester, Leicester, LE1 9HN, UK; 2 Department of Biology, College of Education, University of Mosul, Iraq; 3 Molecular Genetics, University of Groningen, Nijenborgh 7, 9747 AG, Groningen, the Netherlands

## Abstract

Catabolism of galactose by *Streptococcus pneumoniae* alters the microbe’s metabolism from homolactic to mixed acid fermentation, and this shift is linked to the microbe’s virulence. However, the genetic basis of this switch is unknown. Pyruvate formate lyase (PFL) is a crucial enzyme for mixed acid fermentation. Functional PFL requires the activities of two enzymes: pyruvate formate lyase activating enzyme (coded by *pflA*) and pyruvate formate lyase (coded by *pflB*). To understand the genetic basis of mixed acid fermentation, transcriptional regulation of *pflA* and *pflB* was studied. By microarray analysis of Δ*pflB*, differential regulation of several transcriptional regulators were identified, and CcpA, and GlnR’s role in active PFL synthesis was studied in detail as these regulators directly interact with the putative promoters of both *pflA* and *pflB*, their mutation attenuated pneumococcal growth, and their expression was induced on host-derived sugars, indicating that these regulators have a role in sugar metabolism, and multiple regulators are involved in active PFL synthesis. We also found that the influence of each regulator on *pflA* and *pflB* expression was distinct in terms of activation and repression, and environmental condition. These results show that active PFL synthesis is finely tuned, and feed-back inhibition and activation are involved.

Microbes commonly use multiple pathways for metabolism of the same nutrient, very likely due to operation of different pathways, that require distinct environmental conditions^1^,^2^. The presence of functionally similar pathways gives rise to the emergence of different phenotypes. However, the environmental and genetic factors giving rise to different metabolic outcomes, and hence phenotypes, are poorly understood. The important human pathogen *Streptococcus pneumoniae* is a good example for studying this case. It can exist as a commensal in the nasopharynx but also, for incompletely understood reasons, it can be a pathogen, causing diseases with high morbidity and mortality, such as pneumonia, bacteraemia, otitis media and meningitis^3^. It has been demonstrated by several studies that the pneumococcus exhibits different phenotypes in different tissues that enable the microbe to colonize mucosal surfaces or enhance its virulence. Changes in operation of its metabolic machinery under the influence of tissue-specific environmental conditions and nutrients will be a major contributing factor^4^,^5^.

Energetic metabolism has a fundamental influence on pneumococcal colonization and virulence^1^,^2^. *S. pneumoniae* produces its energy exclusively through fermentative breakdown of sugars because it lacks a complete set of genes required for respiration^6^. The pneumococcus has the capacity to utilize 32 different sugars^7^, which may provide a selective advantage to the microbe when it encounters different sugars in host tissues. The two most abundant sugars for the pneumococcus are glucose in the blood and galactose in the respiratory tract^1^,^2^,^8^. Fermentation of each leads to formation of pyruvate, from glycolysis for glucose, and the Leloir or tagatose pathways for galactose^1^. When growing on glucose, homolactic fermentation occurs, with pyruvate being converted to lactic acid by lactate dehydrogenase, thus replenishing NAD^+^. On galactose, however, there is a different metabolic outcome. In this instance, pyruvate can be converted to acetyl-CoA and formate by pyruvate formate lyase (PFL)^2^,^9^. The formation of acetyl-CoA gives the possibility of formation of an additional ATP via the action of acetate kinase or regeneration of two molecules of NAD^+^ with the formation of ethanol.

We show that utilization of galactose leads to the generation of mixed acids, and that pyruvate formate lyase (PFL), converting pyruvate to formate and acetyl-CoA, is the key enzyme for mixed acid fermentation^2^. Mutation of either *pflB*, which codes for PFL, or *pflA*, which codes for the pyruvate formate lyase activating enzyme (PFL-AE), being responsible for posttranslational activation of inactive PFL, results in abrogation of mixed acid fermentation on galactose, and interestingly leads to a decrease in pneumococcal virulence^2^. Despite the importance of PFL activity for pneumococcal metabolism and virulence, transcriptional regulation of the genes responsible for active PFL synthesis is not known in detail, neither in pneumococcus nor in other Gram-positive bacteria. So far, only the catabolite control protein A (CcpA) has been implicated in regulation of *pflB*^10^. However, the mechanism of CcpA-exerted *pflA* and *pflB* regulation remains to be investigated. Moreover, it is reasonable to assume that alternative regulatory systems that are operative independently of CcpA, may also have effect on the regulation of *pflA* and *pflB*^11^.

In this study, our objective was to identify the transcriptional regulators (TR) modulating the expression of genes responsible for active PFL synthesis in *S. pneumoniae*. We found that multiple regulators control the transcription of *pflA* and *pflB*. We gathered evidence showing that some of these regulators are induced by galactose, their control over *pflA* and *pflB* is influenced by sodium formate, they exert regulatory influence on each other, and are required for pneumococcal colonization and virulence.

## Results

### Transcriptome analysis of Δ*pflB*

To identify transcriptional regulators of *pflB*, the transcriptional profile of Δ*pflB* was obtained during growth on galactose relative to the wild type D39 strain. This analysis revealed differential expression of 113 genes in the mutant relative to the wild type (Table 1). The microarray results were validated for selected genes by quantitative real time PCR, and a similar trend of expression was obtained (Table 2). Out of these 113 genes, 62 were down-regulated and 51 were up-regulated in Δ*pflB*. The notable gene classes with differential expression were consistent with PFL’s role in galactose metabolism, pyruvate dissimilation, and catabolic and anabolic reactions, and included those coding for galactose hydrolysis and sugar uptake (n = 27), genes implicated in cell shape (n = 6), and fatty acid biosynthesis and acetate dissimilation (n = 13). Interestingly, the expression of loci containing Leloir pathway genes (n = 7) decreased in the mutant, whereas tagatose pathway genes were not affected, implying that the two galactose catabolic pathways are governed by distinct regulatory processes.

The expression of seven genes annotated as transcriptional regulators was also either significantly up- or down-regulated in the mutant relative to the wild type (Table 1). These were, as annotated in the D39 genome (http://www.ncbi.nlm.nih.gov), MarR (SPD_0379), GlnR (SPD_0447), GntR (SPD_1524), SPD_1594, PlcR (SPD_1745), CcpA (SPD_1797), and NmlR (SPD_1637). These regulators are known to be involved in diverse metabolic functions in bacteria, from the control of sugar metabolism (CcpA), fatty acid synthesis (MarR), control of glutamine metabolism (GlnR), and virulence gene expression (PlcR), to sensing environmental and nutritional cues (GntR)^12^. Differential expression of the seven transcriptional regulators, including CcpA, led to the hypothesis that some of these regulators mediate the expression of *pflA* or *pflB*. We could not test our hypothesis for SPD_1594 because, despite repeated attempts, this gene could not be mutated, implying that it is likely to be essential^13^.

### CcpA, GlnR, and GntR interact with the putative promoters of *pflA* and *pflB*

**Figure 1:**
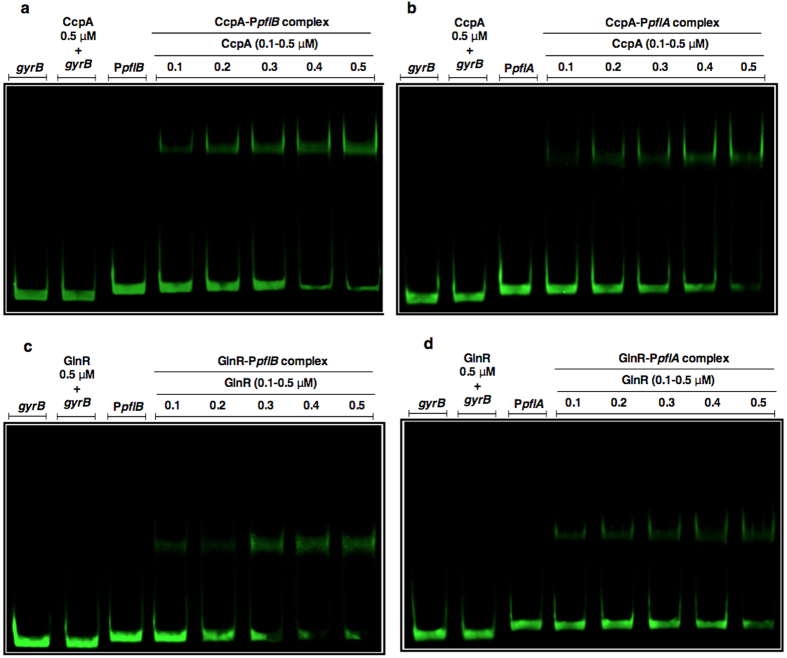
EMSA analysis showing the direct interaction of CcpA (**a** and **b**), GlnR (c and d), with P*pflB* or P*pflA*, respectively. Each lane contains approximately 30 ng P*pflB* or P*pflA*. CcpA and GlnR were used between 0.1 to 0.5 μM. The coding sequence of *gyrB* (30 ng) was used as a negative control. Gels were stained with SYBR Green EMSA for visualizing DNA.

The direct interaction of transcriptional regulators with the putative promoters (P) of *pflA* (P*pflA*) and *pflB* (P*pflB*), was determined by electrophoretic mobility shift assay (EMSA). For this, recombinant CcpA, PlcR, NmlR, GntR, GlnR, and MarR were purified (Supplementary Fig. S1), and their interactions with P*pflA* and P*pflB* were studied. The results showed that, CcpA and GlnR bound to both P*pflA* and P*pflB.* Interaction of transcriptional regulators with their target DNA was concentration-dependent and specific, as the regulators could not bind to a *gyrB*-specific probe (Fig. 1a–d). It should be also noted that CcpA binding to P*pflA* and P*pflB* occurred without the addition of HPr[Ser-P], which is reported to be essential for CcpA-DNA interaction in other lactic acid bacteria^14^,^15^. GntR could also form complexes with P*pflB* (Supplementary Fig. S2) but its inducibility with different sugars, and its involvement in regulation of *pflA* and *pflB* could not be established with subsequent reporter assays. In addition, the other transcriptional regulators did not interact either with P*pflA* or P*pflB* (data not shown).

**Figure 2:**
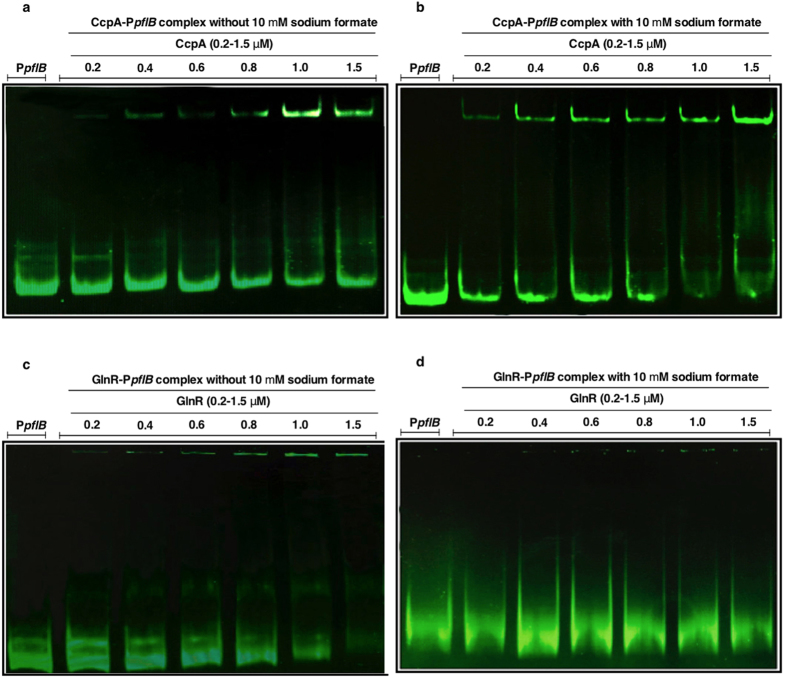
EMSA analysis showing the impact of formate on binding affinity of CcpA and GlnR. Sodium formate enhanced CcpA- and decreased GlnR affinity for P*pflB* (b and d, respectively) compared to CcpA (**a**), and GlnR (**c**) interaction without sodium formate. Each lane contains approximately 30 ng P*pflB*; 0.2–1.5 μM of CcpA, or GlnR with or without 10 mM sodium formate. The experiment was repeated three times, and a representative image is shown.

PFL activity leads to the generation of up to 35 mM formate when the pneumococcus is propagated on 55 mM galactose^2^. Hence, we tested whether sodium formate would alter the binding affinity of CcpA, and GlnR for P*pflB*. For this, the recombinant proteins were preincubated with 10 mM sodium formate before addition of the DNA probe. The results showed that formate enhanced the binding of CcpA to P*pflB* such that with sodium formate Kd was 0.35 ± 0.09 μM, and without it was 1.27 ± 0.1 μM (p < 0.01) (Fig. 2 and Supplementary Fig. S3). The effect of formate was specific because the addition of 10 mM Tris-HCl did not have any effect on binding (data not shown). On the other hand, formate reduced GlnR (Kd: 0.79 ± 0.1 μM) affinity for the *pflB* probe relative to without formate (Kd: 0.31 ± 0.09 μM) (p < 0.01) (Fig. 2).

### CcpA and GlnR bind to promoters containing *cre* sites

After establishing the interaction of CcpA and GlnR with P*pflA* and P*pflB*, we set out to determine the binding site of these regulators. Initially, we mapped the putative binding sites on P*pflA* and P*pflB* (Supplementary Fig. S4), and identified multiple sites resembling a *cre* consensus sequence, the known binding site for CcpA from *Bacillus subtilis* (TGWAARCGYTWNCW, where N is any base, W is A or T, R is A or G, Y is C or T)^16^,^17^. At each putative promoter site, *cre1* had the highest level of homology to the consensus sequence, 12 out of 14 nucleotides being similar. Therefore, EMSA was performed to determine whether *cre1* had any role in binding of CcpA, and GlnR. For this, a promoter probe that excluded the *cre1* sequence was produced and designated as P*pflA(cre1*^−^) and P*pflB(cre1*^−^), which was similar in size, approximately 100 bp, to the P*pflA and* P*pflB*.

**Figure 3:**
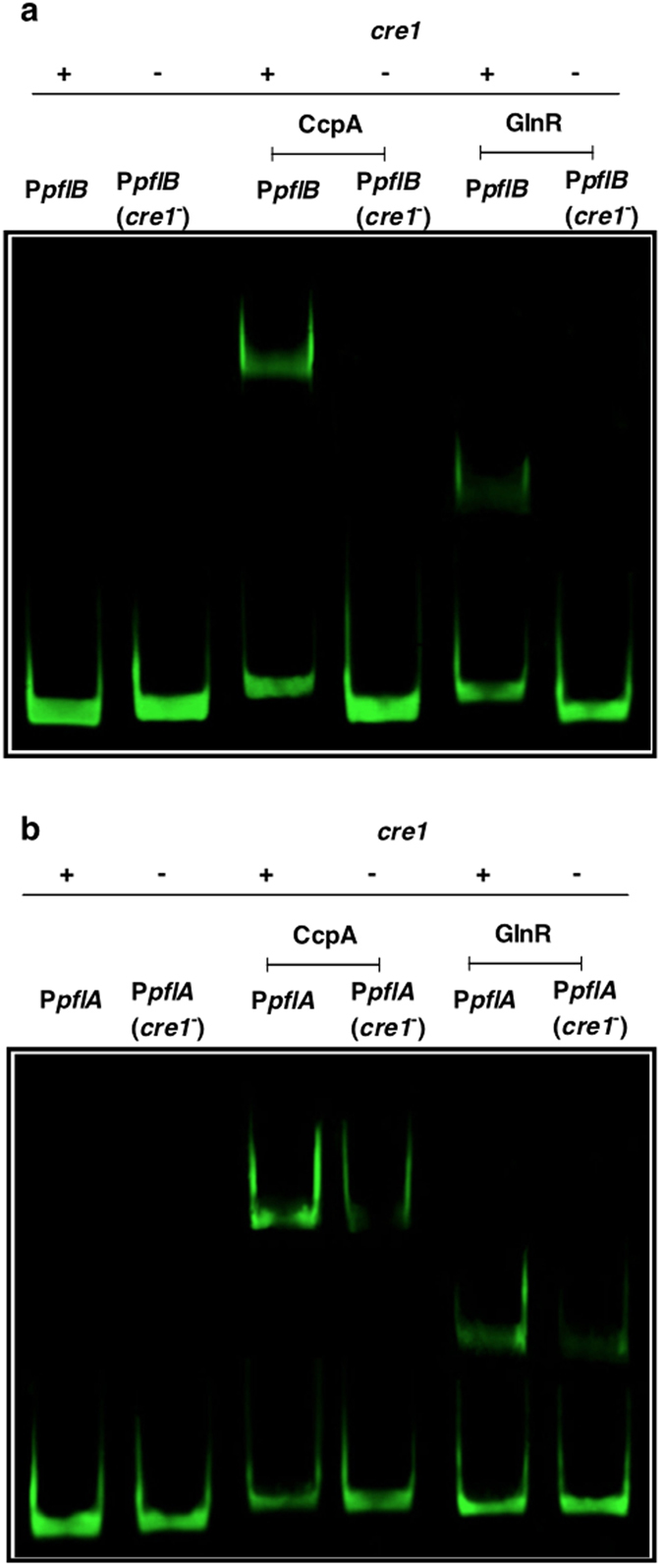
EMSA analysis showing sequence specificity of CcpA, and GlnR for the *cre1* sites in (**a**) Pp*flB* and (**b**) P*pflA*. Each lane contains approximately 30 ng of promoter probes, and 0.5 μM of CcpA or GlnR.

EMSA analysis showed that CcpA and GlnR could not bind to P*pflB(cre1*^−^), indicating that the 14 nucleotide *cre* sequence located in P*pflB* contains the binding site for these regulators (Fig. 3a). Conversely, the results show that CcpA and GlnR bind to both P*pflA* and P*pflA(cre1*^−^) (Fig. 3b), However, they had higher binding affinities for P*pflA* than for P*pflA(cre1*^−^), which has an additional putative *cre*-like sequence.

### Host-derived sugars induce the expression of transcriptional regulators

The inducibility of the regulators by different sugars was determined using transcriptional reporter assays in anaerobically grown cultures. For this, the putative promoter region of each transcriptional regulator was fused to a promoterless *lacZ* in the wild type D39 background, and β-galactosidase activity in resulting reporter strains, P*ccpA*::*lacZ-*wt, and P*glnR*::*lacZ*-wt, was determined in the presence of glucose, galactose, mannose, or *N*-acetyl glucosamine (Table 3). All sugars induced the transcriptional regulator-driven β-galactosidase activity relative to expression in the absence of sugar (p < 0.01), but the highest inducer was found to be galactose.

In addition, the responsiveness of the regulators to 10 mM sodium formate, which does not affect pneumococcal growth, was also determined. For this, the reporter strains were grown in the presence of glucose because formate production was found to be below the detection limit on glucose^2^,^18^, hence endogenous formate would not interfere with the assay. The results showed that the addition of sodium formate, but not Tris-HCl, induced β-galactosidase activity in P*ccpA*::*lacZ-*wt by 2.4-fold, but decreased the activity in P*glnR*::*lacZ*-wt by 4.3-fold compared to the activity on glucose alone (Table 3) (p < 0.01). These data indicate that *ccpA* and *glnR* expression are responsive to formate, and that the absence of formate must be partly responsible for the transcriptional profile of Δ*pflB*.

### CcpA, and GlnR have different roles in expression of *pflA* and *pflB*

**Figure 4:**
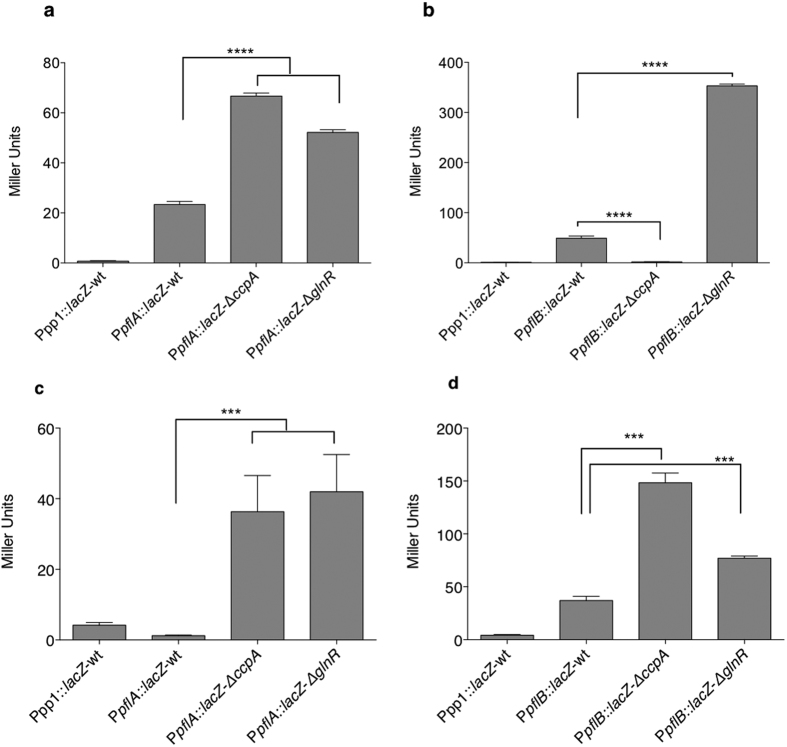
Expression levels (in Miller Units) of pneumococcal transcriptional *lacZ*-fusions to the promoters of *pflA* (**a** and **c**) and *pflB* (**b** and **d**) in different backgrounds grown anaerobically in CDM supplemented with 55 mM of galactose (a and b) or glucose (**c** and **d**). The activity is expressed as nmol *p*-nitrophenol/min/ml. Error bars show the standard error of the mean for three individual measurements each with three replicates. ***p < 0.001, ****p < 0.0001.

To test the regulatory roles of CcpA, and GlnR on *pflA* and *pflB,* P*pflA*::*lacZ* and P*pflB*::*lacZ* fusions were introduced into both wild type D39, and the mutants Δ*ccpA,* and Δ*glnR*. The resulting strains P*pflA*::*lacZ*-wt, P*pflA*::*lacZ-*Δ*ccpA*, P*pflA*::*lacZ-*Δ*glnR*, P*pflB*::*lacZ*-wt, P*pflB*::*lacZ*-Δ*ccpA*, and P*pflB*::*lacZ*-Δ*glnR* were tested during anaerobic growth in CDM supplemented with 55 mM of galactose. The results show that β-galactosidase activity in P*pflA*::*lacZ*-Δ*ccpA* and P*pflA*::*lacZ*-Δ*glnR* increased by 2.8- and 2.2-fold, respectively, compared to the activity in P*pflA*::*lacZ*-wt (Fig. 4a) (p < 0.0001), suggesting that CcpA and GlnR repress the transcription of *pflA*. On the other hand, β-galactosidase activity in P*pflB*::*lacZ*-Δ*glnR* increased by 7.2-fold, whereas in P*pflB*::*lacZ*-Δ*ccpA* it decreased by 28.7-fold compared to the wild type (p < 0.0001) (Fig. 4b), indicating that GlnR repress, and that CcpA activates *pflB* on galactose. On the other hand, on glucose both CcpA and GlnR were found to repress both *pflA* and *pflB*, (Fig. 4c and d) (p < 0.001). To rule out the possibility that the expression data was influenced by *lacZ* fusion, *pflA*, and *pflB* expression was also determined by real time quantitative reverse transcriptase PCR (qRT-PCR) in Δ*ccpA* and Δ*glnR* grown on galactose relative to wild type. The results obtained with qRT-PCR were consistent with those of the transcriptional reporter assays, ruling out any interference by *lacZ* (Supplementary Table S1).

**Figure 5:**
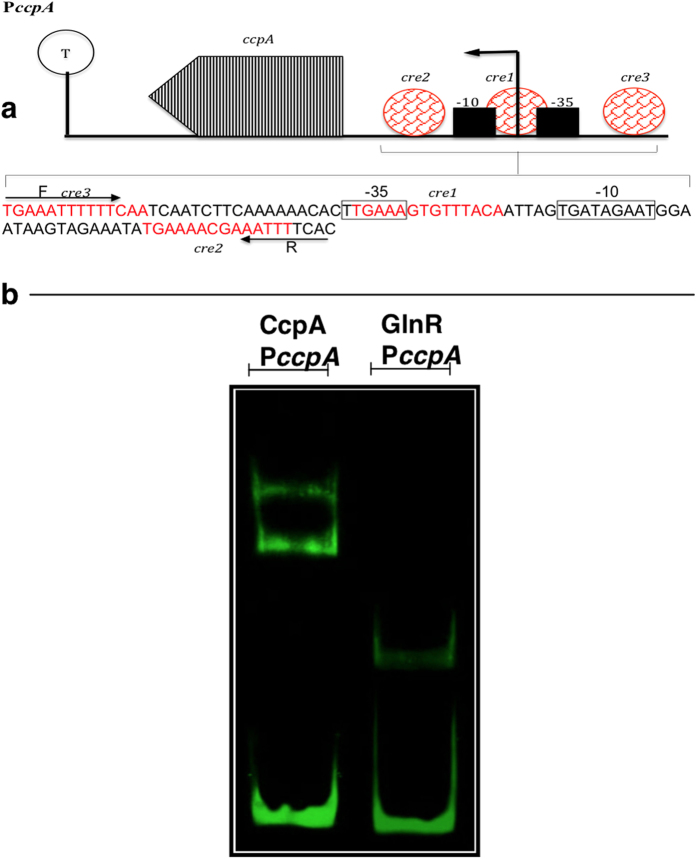
(**a**) Schematic representation showing the analysis of predicted promoter region and binding sites of P*ccpA*, and (**b**) EMSA analysis showing the direct interaction of CcpA and GlnR with P*ccpA*. (**a**) The core promoter region containing the −10 and −35 elements is indicated. The putative *cre* sequences are indicated in red. F: indicates forward primer while R: refers to the reverse primer used for amplifying the promoter probe. T: potential terminator structure. The black arrow presents the direction of transcription. (**b**) Each lane contains approximately 30 ng P*ccpA*; 0.5 μM of CcpA or GlnR as appropriate.

Next, we investigated whether CcpA and GlnR could play a regulatory role in each other’s expression. This hypothesis was tested by use of EMSA and LacZ reporter assays. EMSA showed that both CcpA and GlnR could bind to P*ccpA*, indicating that *ccpA* regulates its own transcription, and that GlnR is involved in *ccpA* expression (Fig. 5). This binding was specific as CcpA and GlnR could not bind to the putative promoter region of *gyrB* (lane 2 in Fig. 1a–d). The interaction between CcpA and GlnR was further investigated by LacZ reporter assays. It was shown that β-galactosidase activity in P*ccpA*::*lacZ*-Δ*ccpA* and P*ccpA*::*lacZ*-Δ*glnR* decreased both on glucose, 2.5- and 10.3-fold, and on galactose, 30.7- and 1.4-fold, respectively, compared to the activity of P*ccpA*::*lacZ*-wt (Table 4) (p < 0.001). This result shows that both on glucose and galactose CcpA increases its own expression, and GlnR activates *ccpA,* particularly on glucose.

CcpA’s role in regulation of *glnR* was also determined. CcpA could not bind to P*glnR*, which was consistent with the lack of a discernible *cre* sequence in the promoter region. The reporter assay results show that β-galactosidase activity in P*glnR*::*lacZ*-Δ*ccpA* decreased by 1.7- and 3.8-fold on glucose and galactose, respectively, compared to P*glnR*-*lacZ*-wt. This result suggests that CcpA increases *glnR* expression in the presence of both glucose and galactose, and GlnR does not effect its own expression, since β-galactosidase activity in P*glnR*::*lacZ*-Δ*glnR* on glucose (89.7 MU ± 4.4, n = 3) and galactose (572.3 MU ± 4.2, n = 3) was similar to that of P*glnR*::*lacZ*-wt (with glucose 93.4 MU ± 1.8, and with galactose: 565.0 MU ± 7.1, n = 3 for both) (Table 4). Moreover, the results showed that GlnR activates *ccpA* expression both on glucose and galactose (Table 4).

### Growth and end product analysis

**Figure 6:**
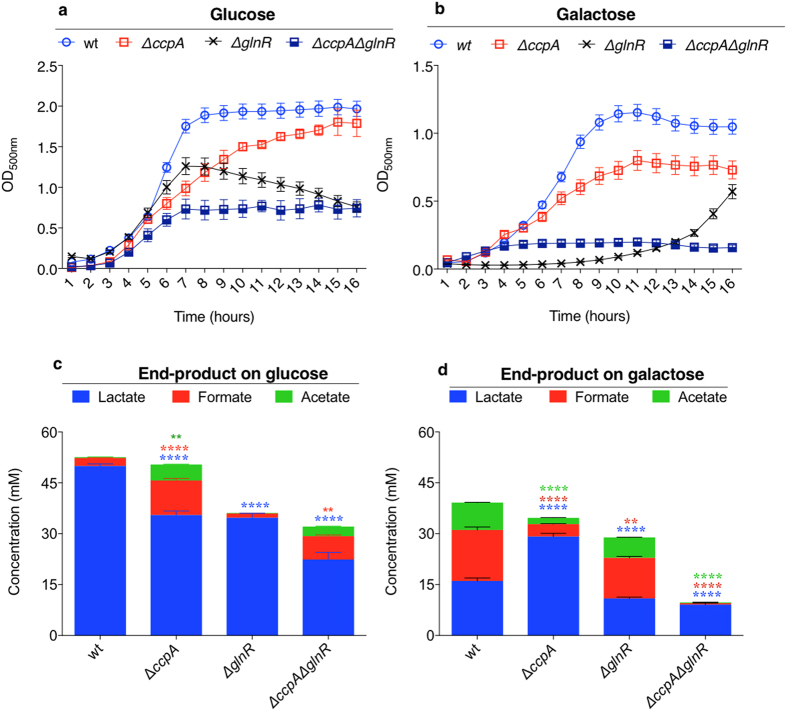
Pneumococcal growth and fermentation end products after culture anaerobically in CDM supplemented with different sugars. (**a**) shows growth in 55 mM glucose and (**b**) in galactose. (**c**) and (**d**) show fermentation end product profiles after growth on glucose and galactose, respectively. Error bars show the standard error of the mean for three individual measurements each with three replicates. Significant differences were seen comparing the growth rates, and the fermentative profile of mutant strains to the wild type D39 using ANOVA followed by Dunnett’s multiple comparison test. **p < 0.01 and ****p < 0.0001.

To further investigate CcpA, and GlnR’s role in pneumococcal sugar metabolism, the growth profiles and fermentative end-products of Δ*ccpA*, and Δ*glnR* were determined. Moreover, to evaluate the added impact of CcpA and GlnR mutation on pneumococcal metabolism, a Δ*ccpA*Δ*glnR* mutant was also constructed and tested. On glucose, Δ*ccpA,* Δ*glnR*, and Δ*ccpA*Δ*glnR* had significantly reduced growth rates (0.36 h^−1^ ± 0.01, 0.47 h^−1^ ± 0.01, and 0.31 h^−1^ ± 0.01 respectively, n = 3) compared to wild type D39 (0.67 h^−1^ ± 0.01) (p < 0.0001 for all strains). Despite a low rate of growth, Δ*ccpA* growth yield (max change in OD500), was similar to that of wild type, while Δ*glnR* had a lower yield than the wild type. In addition, Δ*ccpA*Δ*glnR* had a lower growth rate and yield than each single mutant (p < 0.01) (Fig. 6a). The reason for growth attenuation of strains with a *glnR* mutation, Δ*glnR* and Δ*ccpA*Δ*glnR,* is not known but it may be linked to GlnR’s involvement in glucose transport and metabolism.

On galactose, the growth rate and yield of all strains decreased approximately to half or less than that on glucose (Fig. 6b). Δ*ccpA*, Δ*glnR*, and Δ*ccpA*Δ*glnR* had significantly reduced growth rates (0.24 h^−1^ ± 0.01, 0.26 h^−1^ ± 0.01, and 0.07 h^−1^ ± 0.01, respectively, n = 3) compared to the wild type strain (0.35 h^−1^ ± 0.01, n = 3) (p < 0.01). Δ*glnR* had an extended lag phase and started to grow after 11 h, which may indicate its involvement in sensing of galactose. Moreover, there was significant difference in the rate of growth between Δ*ccpA*Δ*glnR* and each single mutant (p < 0.001), and the growth yield (max change in OD500) of the double mutant (0.21 ± 0.02, n = 3) was significantly lower than that of the single mutants (0.79 ± 0.07 for Δ*ccpA*, 0.74 ± 0.06 for Δ*glnR*, n = 3) (p < 0.01).

In the presence of glucose the wild type, and Δ*glnR* strains displayed typical homolactic behaviour with lactate as the main fermentation product, and minor amounts of formate and acetate (Fig. 6c). Loss of CcpA in Δ*ccpA* and Δ*ccpA*Δ*glnR* caused a shift from homolactic to mixed-acid fermentation. In Δ*ccpA*, a 4.4-fold increase in the yield of formate and 23.5-fold in acetate was detected relative to the wild type. On galactose, the wild type, and Δ*glnR* displayed a mixed-acid fermentation pattern with lactate, formate and acetate as the end products. However, Δ*ccpA* and Δ*ccpA*Δ*glnR* had a homolactic product pattern with lactate production in Δ*ccpA* (29.1 mM ± 0.9, n = 3) being significantly higher than in the wild type (p < 0.0001), whereas formate and acetate production in these strains was significantly decreased compared to the wild type (p < 0.0001) (Fig. 6d). Therefore, the results showed that both transcriptional regulators are important for growth on both glucose and galactose, and CcpA has a major impact on the composition of the mixed acid profile.

### Virulence studies

**Figure 7:**
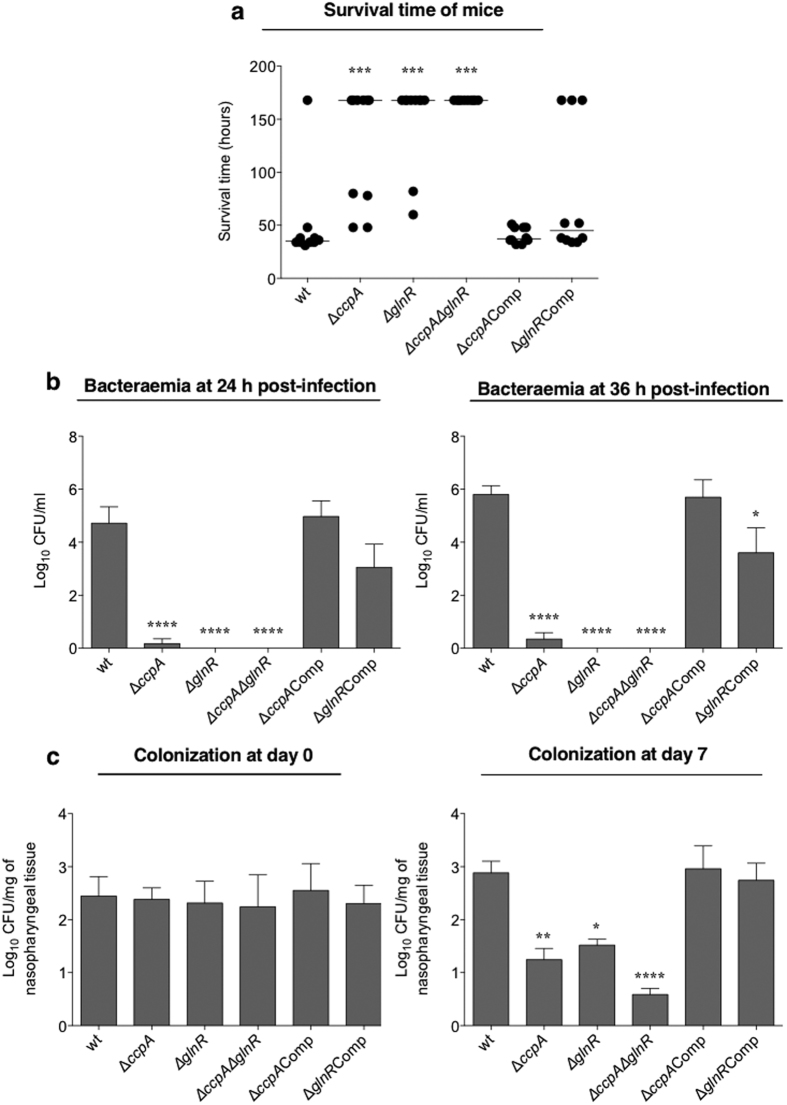
Contribution of CcpA and GlnR to pneumococcal virulence and colonization. (**a**) Survival time of mice infected intranasally with approximately 2 × 10^6^ CFU pneumococci. Each dot represents the survival time of individual animal, and the horizontal bars mark the median survival times derived from 10 animals. (**b**) Progression of bacteraemia in mice infected intranasally with Δ*ccpA*, Δ*glnR* and their derivatives at 24 h and 36 h post-infection. Each point is the mean of data from ten mice. (**c**) Pneumococcal strains defective in *ccpA* and *glnR* were less able to colonize nasopharynx. Mice were infected approximately with 1 × 10^5^ CFU pneumococci. At day 0 and day 7, five mice were culled, and bacterial CFU/mg were determined by serial dilutions of nasopharyngeal homogenates. Each column represents the mean of data from five mice. Error bars show the standard error of the mean. *p < 0.05, **p < 0.01, ***p < 0.001, and ****p < 0.0001.

*In vitro* assays indicated CcpA and GlnR are involved in *pflA* and *pflB* regulation, and they have a regulatory influence on each other’s expression. Therefore, we determined the contribution of CcpA and GlnR to nasopharyngeal colonization, and virulence in mouse models of pneumococcal infection. The results show that the median survival time of mice infected intranasally with Δ*ccpA,* Δ*glnR*, and Δ*ccpA*Δ*glnR* (168 h ± 17.3, 168 h ± 13.0 and 168 h ± 0.0 respectively, n = 10) was significantly longer than the wild type infected group (35 h ± 13.2, n = 10) (p < 0.001 for all strains) (Fig. 7a). The introduction of intact copies of *ccpA* and *glnR* into Δ*ccpA* and Δ*glnR*, respectively, reconstituted the virulence of these strains with the median survival times of mice infected with Δ*ccpA*Comp (37 h ± 2.3, n = 10) and Δ*glnR*Comp (45 h ± 19.5, n = 10), being not significantly different from the wild type infected cohort (p > 0.05), eliminating the possibility of a polar effect of the mutations (Fig. 7a).

The progression of bacteraemia in mice intranasally infected with the pneumococcal strains was also determined. The concentrations of pneumococci retrieved from the blood are shown in Fig. 7b. The bacterial load in the Δ*ccpA* infected group was significantly lower at 24 and 36 h post-infection (log_10_ 0.18 ± 0.18 CFU/ml and log_10_ 0.35 ± 0.2 CFU/ml, respectively, n = 10) compared to the wild type (24 h: log_10_ 4.71 ± 0.6 CFU/ml, 36 h: log_10_ 5.8 ± 0.3 CFU/ml, n = 10) (p < 0.0001). On the other hand, no bacterial growth could be detected in the blood of Δ*glnR* or Δ*ccpA*Δ*glnR* infected animals at 24 and 36 h of infection (p < 0.0001). Moreover, both complemented strains, Δ*ccpA*Comp (24 h: log_10_ 4.97 ± 0.5 CFU/ml, 36 h: log_10_ 5.69 ± 0.6 CFU/ml, n = 10) and Δ*glnR*Comp (24 h: log_10_ 3.05 ± 0.8 CFU/ml, 36 h: log_10_ 3.6 ± 0.9 CFU/ml, n = 10), had significantly higher loads in the blood of infected mice at 24- and 36 h of post infection compared to Δ*ccpA* and Δ*glnR* (p < 0.0001) (Fig. 7b).

We also evaluated the contribution of CcpA and GlnR in nasopharyngeal colonization. One hour after infection of the nasopharynx, the pneumococcal load in the nasopharyngeal tissue for all strains (log_10_ 2.38 ± 0.2 CFU/mg, log_10_ 2.31 ± 0.4 CFU/mg, log_10_ 2.24 ± 0.6 CFU/mg, log_10_ 2.55 ± 0.5 CFU/mg and log_10_ 2.30 ± 0.3 CFU/mg, for Δ*ccpA*, Δ*glnR*, Δ*ccpA*Δ*glnR*, Δ*ccpA*Comp, and Δ*glnR*Comp, respectively, n = 5) was similar to that of wild type (log_10_ 2.44 ± 0.3 CFU/mg, n = 5) (p > 0.05) (Fig. 7c). On the other hand, at 7 days post-infection the colony counts for Δ*ccpA*, Δ*glnR*, and Δ*ccpA*Δ*glnR* (log_10_ 1.24 ± 0.2 CFU/mg, log_10_ 1.52 ± 0.1 CFU/mg and log_10_ 0.58 ± 0.1 CFU/mg respectively, n = 5) were significantly lower than the counts of wild type strain (log_10_ 2.88 ± 0.2 CFU/mg, n = 5) (p < 0.01, p < 0.05 and p < 0.0001 for Δ*ccpA*, Δ*glnR*. and Δ*ccpA*Δ*glnR,* respectively) (Fig. 7c). No significant differences were seen in the bacterial load of the complemented strains, Δ*ccpA*Comp and Δ*glnR*Comp (log_10_ 2.96 ± 0.4 CFU/mg and log_10_ 2.74 ± 0.3 CFU/mg respectively, n = 5) compared to the wild type (p > 0.05). These results show that CcpA and GlnR contribute both to pneumococcal virulence and colonization of nasopharynx.

## Discussion

This study provides new insights into how the important human pathogen *S. pneumoniae* regulates its galactose metabolism, which is known to have a major effect on pneumococcal survival *in vivo*^6^. Pyruvate formate lyase is a key enzyme for pyruvate dissimilation in *S. pneumoniae*. In addition to its role in pneumococcal energetics, the products of the PFL-catalysed reaction, formate, and acetyl CoA, play a key role in anabolic reactions. For example, while formate is essential for serine biosynthesis by hydroxymethylation of glycine, acetyl-CoA is important for fatty acid biosynthesis^2^,^19^. Until this study, very little was known about the transcriptional regulation of genes involved in active PFL synthesis.

Our results show that multiple regulators modulate the expression of *pflA* and *pflB*. While CcpA and GlnR are involved in regulation of both *pflA* and *pflB*, GntR could only bind to putative promoter region of *pflB*. The other regulators identified to be differentially expressed in Δ*pflB* could bind neither to P*pflA* nor to P*pflB*, presumably their differential expression in Δ*pflB* was due to indirect effect of *pflB* mutation. CcpA was found to repress *pflA* and *pflB* on glucose, and activate *pflB* and repress *pflA* expression on galactose. This opposing regulatory influence of CcpA on *pflA* and *pflB* could indicate that constitutive *pflA* expression sufficiently activates PFL synthesis. This is supported with the fact that the association rate between PFL-AE and PFL is low for biological interactions because of large conformational changes when these two enzymes interact. Hence a high level *pflA* expression would be wasteful because of a low association rate^20^. Moreover, intracellular calculations in *E. coli* revealed that PFL-AE is almost entirely in the PFL-AE/AdoMet PFL pyruvate complex, and its activation requires reduction by flavodoxin to initiate catalysis. Therefore, despite its constitutive expression, the existing PFL-AE can be promptly activated in this complex when needed. It was also noticed that induction level of P*pflA* is lower than P*pflB* on galactose (Fig. 4). The expression data are very likely reflecting the amount of PFL-AE required to activate PFL, because PFL is completely activated by 0.01 equivalents of PFL-AE in 100 minutes^21^. The opposing effects of transcriptional regulators on *pflA* and *pflB* must be an important pneumococcal adaptation mechanism in tissues with different sugar profiles and oxygen concentrations. For example, in an oxygenated environment active PFL is not critical as it is sensitive to oxygen. On the other hand, in tissues with high galactose content but limited oxygen concentration, the pneumococcus requires mechanisms to induce *pflB* expression.

In other Gram positive bacteria CcpA binding requires HPr-[Ser-P], while the pneumococcal CcpA bound to a *cre* sequence in the absence of HPr-[Ser-P]^22^. However, we found that 10 mM sodium formate enhanced CcpA affinity for P*pflB*, consistent with its induction by sodium formate in the transcriptional reporter assays. Although the inducibility of *ccpA* expression by different carbon compounds had been shown previously^11^,^23^, this is the first demonstration of the responsiveness of this master regulator to formate. It is not clear how formate enhances CcpA binding and stimulates the expression, but given that formate is a potent reducing agent, with a redox potential of −420 mV^24^, it is very likely due to its effect on the conformation or multimerization of protein structure. Addition of formate increased *ccpA*-, and decreased *glnR* transcription, indicating the responsiveness of *S. pneumoniae* to formate. Formate’s role as a transcriptional signal also shows that the absence of formate is partly responsible for the transcriptional profile of Δ*pflB.* In addition, we previously demonstrated that fatty acid composition of *S. pneumoniae* changes in Δ*pflB*, which cannot produce formate, also indicating the importance of formate on transcription and protein synthesis^2^. Formate was reported to have a major influence on bacterial gene expression, and acts as a diffusible signal to induce *Salmonella enterica* serovar Typhimurium invasion^25^. In *Staphylococcus aureus*, it was suggested that formate production is necessary for formyl-tetrahydrofolate synthesis, which is required for protein and purine synthesis^18^. Further work is required to understand the mechanism by which formate impacts on the pneumococcus. We did use galactose similarly to formate to determine its effect on the affinity of transcriptional regulators to P*pflA* and P*pflB*. However, no difference could be detected.

The *cre1* within P*pflB* has been identified to be the binding site for GlnR. GlnR bound two *cre* sites in P*pflA* but here it had a higher binding affinity for *cre1* than *cre2*. GlnR binding affinity for *pflB* was shown to decrease in the presence of 10 mM sodium formate, consistent with a decrease in P*glnR* driven β-galactosidase activity with sodium formate. This is the first demonstration of interaction of proteins, other than CcpA and HPr, with *cre*. Given that *cre* sites are plentiful in Gram-positive bacteria, it is plausible that some of these targets are regulated by multiple regulators, either independently or in concert with CcpA.

Interestingly, although CcpA plays a role in *glnR* regulation, as determined by the LacZ reporter assay, P*glnR* does not have an identifiable *cre*-like sequence. Therefore, it is possible that CcpA-mediated *glnR* regulation requires the involvement of another, unknown, protein(s). It is known that several genes with no *cre* or un-functional *cre* have been reported to be indirectly regulated by CcpA^26^,^27^,^28^. Our results showed that both CcpA and GlnR have a significant role in pneumococcal virulence and colonization. The results obtained in this study on CcpA’s impact on pneumococcal colonization and virulence are in line with previous reports^11^,^23^. However, our *in vivo* results for GlnR differ from the study of Hendriksen *et al*., who could not attribute a role for GlnR in pneumococcal virulence and colonization^29^. One reason for this discrepancy can be linked to the difference in dose-preparation, for example, while in this study we did not passage the bacterial inoculum through peritoneum before infection, Hendriksen’s study used passaged bacteria. *In vivo* passaging before infection can introduce mutations to a bacterial population due to strong within-host selective pressure^30^.

The GntR family of transcriptional regulators is a group of transcriptional repressors distributed among diverse bacterial groups, and mediate bacterial response to nutritional and/or environmental signals^31^. In addition to CcpA and GlnR, GntR could also bind to P*pflB*. However, GntR could not bind to P*ccpA* and P*pflA*, even though both have a *cre*-like sequence. Unlike *cre1* in P*pflB* (TGAAATCGGTTACT, conserved residues are underlined), which is categorised as a high-affinity *cre* box because it contains all four strongly conserved residues, G2, C7, G8, and C13, the *cre* sequences in P*pflA*, and P*ccpA* contain only 2 or 3, respectively, conserved residues^16^,^17^. Therefore, it is likely that GntR only recognizes highly conserved *cre* boxes. P*gntR*-driven β-galactosidase activity was not induced by any of the tested sugar nor Δ*gntR* is attenuated in growth on galactose (data not shown). This suggests that in addition to sugar dependent regulation, sugar independent regulation of *pflA* and *pflB* may take place.

**Figure 8:**
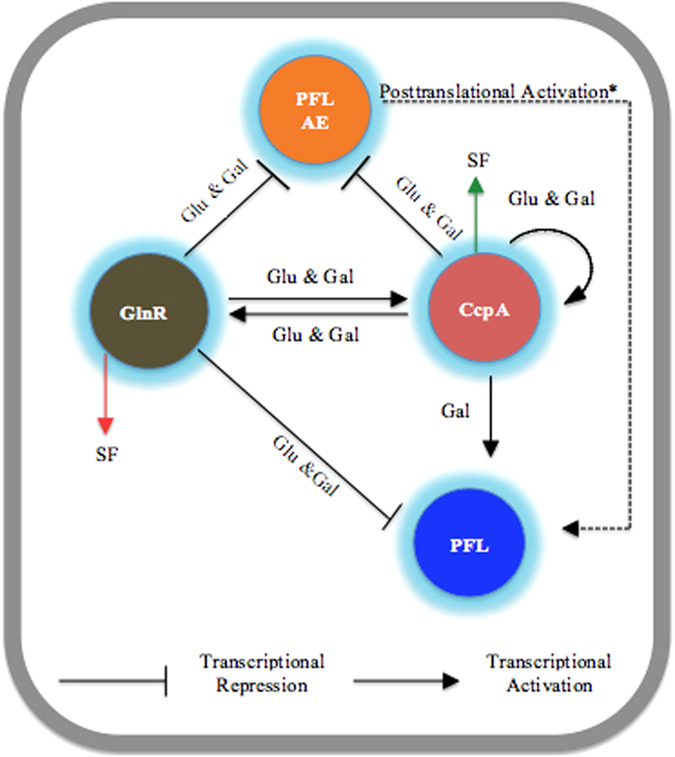
Schematic model of *pflB* and *pflA* regulation. On galactose, formate production by PFL activity decreases GlnR affinity for P*pflB* and GlnR increases *ccpA* transcription. The increased formate production increases CcpA affinity for P*pflB*, and CcpA either alone or after interacting with GlnR binds to P*pflB* and increases *pflB* transcription. However, CcpA also binds to P*pflA* and represses its expression to fine-tune the level of active PFL. Increased formate production then creates a positive feed back loop, and CcpA self-regulates its own expression. Glu: glucose, Gal: galactose, SF: sodium formate. The green arrow indicates increase in expression, whereas the red arrow is for decreased expression. *Posttranslational activation of PFL by PFL-AE has been previously reported^9^,^47^.

Based on the available data we propose the model for *pflA* and *pflB* regulation shown in Fig. 8. Growth on galactose increases PFL activity, which leads to increased production of formate, and also induces *glnR* expression. When the formate level reaches a certain threshold, GlnR repression over *pflA* and *pflB* expression is removed, and GlnR then binds to *cre,* up-regulating *ccpA* transcription. In addition, the increased formate production also increases *ccpA* expression as well as CcpA affinity for P*pflB*, leading to an increase in *pflB* transcription. However, CcpA and GlnR also bind to P*pflA,* and repress *pflA* expression to fine-tune the level of *pflA* transcript. On the other hand, on glucose, GlnR increases *ccpA* expression, and CcpA activates its own expression. This, in turn, results in repression of both *pflA* and *pflB*, and pyruvate is dissimilated mainly to lactate by lactate dehydrogenase activity^1^,^2^.

Galactose is found in high concentration on host glycoconjugates in the nasopharynx, where the pneumococcus resides, and the microbe is able to cleave and utilize galactose from these sources^8^. However, galactose is not efficiently catabolized by the pneumococcus, possibly due to the possession of low-affinity galactose importers^6^, and/or to a slow association rate between nascent PFL and PFL-AE for the formation of active PFL^32^. In addition, at the transcriptional level the expression of *pflA* and *pflB* is down-regulated by the regulators identified in this study, with the exception of CcpA, in the presence of galactose. Taken together, these data indicate that the pneumococcus has evolved to catabolize galactose at a lower rate than other sugars, such as glucose. It can be speculated that low galactose catabolic rate allows the microbe to maintain a stable relationship with its host. This can be linked to galactose catabolism and its role in pneumococcal virulence. For example, the pneumococcus produces more capsule, an essential virulence determinant, on galactose than it does on glucose^10^.

This study demonstrates that PFL synthesis is fine-tuned at the transcriptional level by multiple regulators and various environmental conditions. PFL activity is known to be crucial for galactose catabolism, which is linked to pneumococcal colonization and virulence. Detailed knowledge of regulation of pneumococcal sugar metabolism may allow identification of targets for new antiinfectives against this important pathogen.

## Materials and Methods

### DNA manipulations

Standard methods were used for chromosomal DNA isolation, restriction enzyme digestion, cloning, transformation, agarose gel electrophoresis, and sodium dodecyl sulphate polyacrylamide gel electrophoresis^33^. Plasmids were extracted using QIAprepSpin Miniprep Kit (Qiagen, UK), and PCR products were purified using the Wizard^®^ SV Gel and PCR Clean-Up System (Promega, UK).

### Bacterial strains, plasmids and growth conditions

The strains and plasmids used and constructed in this study are listed in Supplementary Table S2. Pneumococcal strains were grown microaerobically at 37 °C as described previously^2^. For anaerobic growth, an anaerobic cabinet was used. Pneumococci were also grown in chemically defined medium with selected sugars (CDM)^1^. Growth was monitored by measuring the optical density at 500 nm (OD500). Growth rates (μ) were calculated through linear regressions of the plots of ln(OD500) versus time during the exponential growth phase. When required, the pneumococcal growth medium was supplemented with spectinomycin (100 μg/ml), tetracycline (15 μg/ml), and kanamycin (250 μg/ml).

*Escherichia coli* strains, used for cloning and protein expression, were grown in Luria Bertani (LB) broth with shaking at 37 °C or on LB agar plates. For *E. coli* cultures, ampicillin and kanamycin at 100- and 50 μg/ml, respectively, were used when required.

### Microarray analysis

*S. pneumoniae* D39 and its isogenic mutant strain Δ*pflB*, deficient in pyruvate formate lyase (PFL) activity, were grown anaerobically in CDM supplemented with 55 mM galactose as the main carbon source. The experiments were repeated with four biological replicates. The *MicroPrep* software package was used to obtain the microarray data from the slides. CyberT implementation of a variant of *t-test* (http://bioinformatics.biol.rug.nl/cybert/index.shtml) was performed and false discovery rates (FDRs) were calculated^27^. For differentially expressed genes, p < 0.001 and FDR < 0.05 were taken as standard. For the identification of differentially expressed genes a Bayesian p-value of < 0.001 and a fold change cut-off twofold were applied. All other procedures for the DNA microarray experiments and data analysis were performed as described before^34^.

### Gene splicing by overlap extension (gene SOEing)

Allelic replacement mutagenesis was achieved by transformation with an *in vitro* mutagenized SOEing construct. To do this, spectinomycin (Spec^R^) or kanamycin (Kan^R^) resistance gene cassettes^35^,^36^ were amplified using the primes Spec-F and Spec-R or Kan-F and Kan-R primers (Supplementary Table S3). The primers LF-SOEX-F/LF-SOEX-R and RF-SOEX-F/RF-SOEX-R (where X indicates the gene code) were used to amplify the left and right flanking regions of each target gene, generating PCR products of approximately 600 bp in length. The amplified antibiotic resistance cassette and the PCR products flanking the target gene were then fused using LF-SOEX-F and RF-SOEX-R primers. The fused product was gel-purified after electrophoresis (Qiagen) and transformed into *S. pneumoniae*^37^. The transformants were selected on blood agar base supplemented with 5% (v/v) defibrinated horse blood containing the appropriate antibiotic and the mutations were confirmed by PCR and sequencing. The double mutant was constructed by transformation of amplicons representing the mutated region of *glnR* into the *ccpA* mutant.

### Genetic complementation of mutants

To eliminate the possibility of polar effects, the mutant strains were genetically complemented using plasmid pCEP^36^. The wild type copy of mutated genes with their upstream sequence were amplified with X-Comp-F and X-Comp-R primers (where X indicates the gene code) (Supplementary Table S3). The amplicons were digested with *NcoI*-*BamHI* or *SphI*-*BamHI* and were ligated into similarly digested pCEP. A sample of ligation mixture was transferred into One Shot^®^ TOP10 competent *E. coli* cells (Invitrogen, UK). The transformants were selected on kanamycin-containing LB agar plates, and successful cloning was confirmed by PCR using Comp-Seq-F and Comp-Seq-R primers. The recombinant plasmid was purified, and a portion was transformed into the appropriate mutant strains. The complemented strains were designated as Δ*ccpA*Comp, and Δ*glnR*Comp.

### Quantification of extracellular metabolites

Samples (2 ml) of anaerobic cultures growing in CDM containing 1% (w/v) glucose or galactose were collected at late exponential phase during growth, centrifuged (6700 × g, 10 min, 4 °C), filtered (Millex-GN 0.22 μm filters) and the supernatant solutions were stored at −20 °C until analysis with commercial kits for lactate, formate, and acetate detection (Megazyme, Ireland). The amount of organic acids produced by different strains was normalized to 1 × 10^8^ cells. The spent culture supernates were obtained at late exponential phase and this corresponded to the following OD500s for the different strains grown on glucose and galactose, respectively: for D39 1.9 and 1.1; for Δ*ccpA* 1.8 and 0.7; for Δ*glnR* 1.2 and 0.6; for Δ*ccpA*Δ*glnR* 0.7 and 0.2.

### Purification of recombinant proteins

The genes of interest were amplified and cloned into the hexa histidine-tagged, ampicillin resistant plasmid pLEICS-01 (PROTEX, University of Leicester) using the primers (Indicated with C-F/R in Supplementary Table S3) containing 15 nucleotides complementary to the cloning site in pLEICES-01. Recombinant plasmids were transformed into *E. coli* BL21 (DE3) pLysS (Agilent Tech, USA) for protein expression at 18 °C and 0.1 M of IPTG (Isopropyl β-D-1-thiogalactopyranoside) was used for induction of protein expression. When the OD600 reached 1.5–1.6, the culture was centrifuged and the pellet was resuspended with the binding buffer (20 mM Tris, 150 mM NaCl, pH 7.45) prior to sonication at an amplitude of 8 microns for 15 sec with 45 sec intervals on ice. The lysate was then centrifuged and filtered through a 0.45 μm acrodisc (Fisher Scientific, UK). Proteins were purified using PD-10 columns (GE Healthcare Life Sciences, UK) containing 2 ml of TALON Metal Affinity Resin (Clontech, USA) and finally eluted using different concentrations of imidazole elution buffer (20 mM Tris, 150 mM NaCl, pH 7.45). Protein fractions were dialysed against the binding buffer using Amicon Ultra-15 Centrifugal Filter Units (Millipore, UK) and the concentrations were determined using the Bradford protein assay^38^.

### Electrophoretic mobility shift assay (EMSA)

*In silico* analysis, using the bacterial promoter prediction tool (BPROM)^39^ and the Motif-based sequence analysis tools (MEME), was used to predict the presence of regulatory elements in the putative promoter regions of target genes^40^. Based on *in silico* analysis, primers (indicated by EMSA-F/R in Supplementary Table S3) were designed to amplify 95–100 bp DNA fragment representing putative promoter sites upstream of each gene.

EMSA was performed according to the protocol described previously^41^ using Molecular Probes fluorescence-based EMSA kit (Invitrogen). Briefly, 5X FY binding buffer (20 mM Tris-HCl pH 7.5, 30 mM KCl, 1 mM DTT, 1 mM EDTA pH 8.0 and 10% v/v glycerol) was prepared to incubate the promoter probes and proteins. The binding reaction was set up by mixing a constant amount of target promoter probes (~30 ng), and increasing amounts (0.1–0.5 μM) of purified and dialysed His-tagged proteins. The binding reaction was incubated at room temperature for 20 min in a total volume of 20 μl, and then analysed on an 8% w/v non-denaturing polyacrylamide gel. After electrophoresis, gels were stained with SYBR^®^ Green EMSA gel stain (Invitrogen) and visualized using a Typhoon Trio^+^ scanner (GE Healthcare Life Sciences) with a 526 nm short-pass wavelength filter.

### Construction of *lacZ*-fusions

Chromosomal transcriptional *lacZ*-fusions to the target promoters were constructed via double crossover in the *bgaA* gene by using the integration plasmid pPP1^42^. As the extracellular BgaA enzyme is responsible for removal of galactosides from complex carbohydrates, inactivation of the *bgaA* gene will not change the growth of *S. pneumoniae* in media and will not compromise regulatory studies^42^. The putative promoter regions were amplified using the primers modified to incorporate *SphI* and *BamHI* sites (indicated with Fusion-F/R in Supplementary Table S3). The amplicons were digested with *SphI* and *BamHI* and were ligated into similarly digested pPP1. All plasmid constructs were confirmed by sequencing.

### β-galactosidase assays

β-galactosidase activity was measured as described before^43^ using cells grown anaerobically in CDM at 37 °C supplemented with 55 mM of the selected sugars, or 10 mM sodium formate, and harvested in the mid-exponential phase of growth.

### Total RNA purification and quantitative RT-PCR

The extraction of RNA was done by the Trizol method using mid-exponential phase cultures, as described previously^44^. Before use the RNA was treated with DNase using a TURBO DNA-free™ Kit (Ambion, UK), and subsequently purified with an RNeasy Mini Kit (Qiagen).

First strand cDNA was synthesized using approximately 1 μg of DNase-treated total RNA, immediately after isolation, random hexamers and 200 U of SuperScript III reverse transcriptase (Invitrogen) at 42 °C for 55 min as described previously^2^. Three independent RNA preparations were used for qRT-PCR analysis. The transcription level of specific genes was normalized to *gyrB* transcription, which was amplified in parallel using the primers in Supplementary Table S4 (indicated with RT-F/R tags). cDNA samples obtained from individual biological samples were analysed in triplicates. The results were analysed by the comparative threshold cycle C_T_ method^45^. Differences in expression of twofold or greater relative to control were considered as significant.

### *In vivo* virulence studies

To evaluate the virulence of pneumococcal strains, nine to ten-week-old female MF1 outbred mice (Charles River, UK) were lightly anesthetized with 3% v/v isoflurane and oxygen mixture, and an inoculum of 50 μl containing approximately 2 × 10^6^ CFU in PBS was administered dropwise into the nostrils. Disease signs in infected animals were monitored regularly^2^,^46^. When the mice become lethargic, they were culled by cervical dislocation, “survival time” was defined as the time to reach the lethargic state. Mice that were alive 7 days after infection were deemed to have survived the infection. To monitor the development of bacteraemia, approximately 20 μl of venous blood was obtained from each mouse at predetermined time points after infection, and viable counts were determined, as described above. Colonization experiments were done principally as described previously, using 5 × 10^5^ CFU of *S. pneumoniae* in 10 μl PBS, given intranasally^8^. Survival data were analysed by the Mann-Whitney test. Colonization and bacteraemia data were analysed by analysis of variance followed by Tukey’s multiple comparisons test. Statistical significance was considered to be a p*-*value of < 0.05.

### Ethics statement

Mouse experiments at the University of Leicester were performed under appropriate project (permit no. 60/4327) and personal (permit no. 80/10279) licenses according to the United Kingdom Home Office guidelines under the Animals Scientific Procedures Act 1986, and the University of Leicester ethics committee approval. The protocol was approved by both the U.K. Home Office and the University of Leicester ethics committee. Where indicated, the procedures were carried out under anesthetic with isoflurone. Animals were housed in individually ventilated cages in a controlled environment, and were frequently monitored after infection to minimize suffering. Every effort was made to minimize suffering and in bacterial infection experiments mice were humanely culled if they became lethargic.

### Data Availabilty

Microarray data have been submitted to GEO (Gene Expression Omnibus) database under the accession number GSE67668 (https://www.ncbi.nlm.nih.gov/geo/query/acc.cgi?token=ypkhqogmvhwrvst&acc=GSE67668).

## Additional Information

**How to cite this article**: Al-Bayati, F. A. Y. *et al*. Pneumococcal galactose catabolism is controlled by multiple regulators acting on pyruvate formate lyase. *Sci. Rep.* **7**, 43587; doi: 10.1038/srep43587 (2017).

**Publisher's note:** Springer Nature remains neutral with regard to jurisdictional claims in published maps and institutional affiliations.

## Supplementary Material

Supplementary Information

## Figures and Tables

**Table 1:** Microarray analysis of gene expression in ∆*pflB* relative to wild type D39 grown anaerobically in CDM supplemented with galactose.

GeneID	Function	Ratio^a^
SPD_0065	Beta-galactosidase 3	3.2
SPD_0066	PTS system, IIB component	2.6
SPD_0067	PTS system, IIC component	2.4
SPD_0068	PTS system, IID component	2.7
SPD_0069	PTS system, IIA component	1.9
SPD_0094	Hypothetical protein	2.3
SPD_0095	Hypothetical protein	2
SPD_0128	MutT/nudix family protein	2.3
SPD_0145	Hypothetical protein	2
SPD_0148	Ransporter, major facilitator family protein	2.8
SPD_0149	Hypothetical protein	2.2
SPD_0308	ATP-dependent Clp protease, ATP-binding subunit, putative	2
SPD_0423	ROK family protein	2
SPD_0447	Transcriptional regulator, MerR family	2.4
SPD_0448	Glutamine synthetase, type I glnA	2
SPD_0459	Heat shock protein GrpE	3
SPD_0687	ABC transporter, ATP-binding protein	2.9
SPD_0688	Hypothetical protein	2.9
SPD_0915	Iron-compound ABC transporter, iron compound-binding protein	2
SPD_0918	Iron-compound ABC transporter, ATP-binding protein	2
SPD_1375	Hypothetical protein	2.3
SPD_1524	Transcriptional regulator, GntR family	4.1
SPD_1525	ABC transporter, ATP-binding protein	2.2
SPD_1588	Hypothetical protein	2.1
SPD_1589	Hypothetical protein	3
SPD_1590	General stress protein 24, putative	2.6
SPD_1591	Hypothetical protein	2.2
SPD_1594	Transcriptional regulator	2.7
SPD_1595	Hypothetical protein	2.7
SPD_1644	Hypothetical protein	2.1
SPD_1744	Hypothetical protein	2.5
SPD_1745	Transcriptional regulator PlcR, putative	7.1
SPD_1746	Hypothetical protein	13
SPD_1749	Bacteriocin formation protein, putative	9.4
SPD_1750	Hypothetical protein	11.5
SPD_1751	Hypothetical protein	8.1
SPD_1752	Toxin secretion ABC transporter, ATP-binding/permease protein	10.2
SPD_1754	Hypothetical protein	5.5
SPD_1755	Hypothetical protein	5.4
SPD_1756	ABC transporter, ATP-binding protein	3.8
SPD_1756	Hypothetical protein	2.5
SPD_1830	Glycosyl hydrolase, family 1	2
SPD_1831	PTS system, IIC component	2.8
SPD_1832	PTS system, IIB component	2.2
SPD_1943	Hypothetical protein	2.1
SPD_1944	Secreted 45 kd protein	4
SPD_1945	Rod shpae-determining protein MreD, putative	4.6
SPD_1946	Rod shape-determining protein MreC	4.9
SPD_1947	Hypothetical protein	5.5
SPD_1948	ABC transporter, ATP-binding protein	3.7
SPD_1962	Hypothetical protein	11.4
SPD_0116	Hypothetical protein	−3.9
SPD_0161	Hypothetical protein	−2.6
SPD_0262	PTS system, mannose/fructose/sorbose family protein, IID component	−2.3
SPD_0263	*manM* PTS system, mannose-specific IIC component	−2
SPD_0264	*manL* PTS system, mannose-specific IIAB components	−1.7
SPD_0378	enoyl-CoA hydratase	−2.7
SPD_0379	Transcriptional regulator, MarR family	−2.3
SPD_0380	3-oxoacyl-(acyl carrier protein) synthase	−2.4
SPD_0381	Acyl carrier protein	−2.5
SPD_0382	enoyl-(acyl-carrier-protein) reductase	−3.3
SPD_0383	Acyl-carrier-protein S-malonyltransferase	−3.6
SPD_0384	3-ketoacyl-(acyl-carrier-protein) reductase	−4.3
SPD_0385	3-oxoacyl-(acyl carrier protein) synthase	−5.2
SPD_0386	Acetyl-CoA carboxylase	−4.3
SPD_0387	(3 R)-hydroxymyristoyl ACP dehydratase	−4.6
SPD_0388	Acetyl-CoA carboxylase	−6.3
SPD_0389	Acetyl-CoA carboxylase beta subunit	−5.4
SPD_0390	Acetyl-CoA carboxylase alpha subunit	−5.6
SPD_0407	Hypothetical protein	−2
SPD_0420	Formate acetyltransferase	−2.9
SPD_0471	Pseudo	−2
SPD_0511	5,10-methylenetetrahydrofolate reductase	−2.4
SPD_0652	Branched-chain amino acid ABC transporter, amino acid-binding protein	−1.6
SPD_0653	Branched-chain amino acid ABC transporter, permease protein	−1.9
SPD_0654	Branched-chain amino acid ABC transporter, permease protein	−2.3
SPD_0655	Branched-chain amino acid ABC transporter, ATP-binding protein	−2.7
SPD_0656	Branched-chain amino acid ABC transporter, ATP-binding protein	−2.5
SPD_0727	Hypothetical protein	−2.8
SPD_0728	Hypothetical protein	−2.2
SPD_0729	hemolysin-related protein	−2
SPD_0730	deoD purine nucleoside phosphorylase	−2.5
SPD_0751	Hypothetical protein	−2.3
SPD_0752	Hypothetical protein	−2.9
SPD_0753	pcp pyrrolidone-carboxylate peptidase	−2.9
SPD_0761	Hypothetical protein	−2.1
SPD_0997	hup DNA-binding protein HU	−2.1
SPD_1016	Serine/threonine protein phosphatase	−2
SPD_1101	Signal recognition particle-docking protein FtsY	−1.9
SPD_1102	Cof family protein	−2
SPD_1103	Cof family protein	−1.7
SPD_1301	NADPH-dependent FMN reductase	−2.2
SPD_1302	Oxidoreductase, putative	−2.2
SPD_1360	Hypothetical protein	−2.1
SPD_1514	ABC transporter, ATP-binding protein	−2
SPD_1515	Hypothetical protein	−2.1
SPD_1632	Hypothetical protein	−3.3
SPD_1633	*galT*−2 galactose-1-phosphate uridylyltransferase	−2.9
SPD_1634	*galK* galactokinase	−3.4
SPD_1635	*galR* galactose operon repressor	−1.9
SPD_1636	Alcohol dehydrogenase, zinc-containing	−2.8
SPD_1637	Transcriptional regulator, MerR family protein	−3.2
SPD_1638	Cation efflux system protein	−3.4
SPD_1667	Oligopeptide ABC transporter, ATP-binding protein AmiF	−2.4
SPD_1668	Oligopeptide ABC transporter, ATP-binding protein AmiE	−2.3
SPD_1669	Oligopeptide ABC transporter, permease protein AmiD	−2
SPD_1670	Oligopeptide ABC transporter, permease protein AmiC	−1.6
SPD_1671	Oligopeptide ABC transporter, oligopeptide-binding protein AmiA	−1.5
SPD_1707	Hypothetical protein	−2.5
SPD_1797	*ccpA* catabolite control protein A	−2.1
SPD_1965	Choline binding protein PcpA	−5.8
SPD_2021	Glycerol uptake facilitator protein	−2.1
SPD_2033	Ribosomal subunit interface protein	−2.3

^a^Ratios ≥2 or ≤−2.0 (∆*pflB* compared with D39 wild type).

All P-values are <0.001.

**Table 2:** Fold difference in expression of genes for transcriptional regulators in *S. pneumoniae* Δ*pflB* relative to wild type D39 strain grown anaerobically in CDM supplemented with galactose^a^.

GeneID/symbol	Function	Microarray	qRT-PCR
SPD_1797 (*ccpA*)	Catabolite control protein A (CcpA)	−2.1	−2.1 ± 0.03
SPD_1745 (*plcR*)	Transcriptional regulator, PlcR family	7.1	2.0 ± 0.05
SPD_1637 (*nmlR*)	Transcriptional regulator, MerR family	−3.2	−2.2 ± 0.05
SPD_1594	Transcriptional regulator	2.7	2.6 ± 0.1
SPD_1524 (*gntR*)	Transcriptional regulator, GntR family	4.1	2.0 ± 0.1
SPD_0447 (*glnR*)	Transcriptional regulator, MerR family	2.4	2.1 ± 0.09
SPD_0379 (*marR*)	Transcriptional regulator, MarR family	−2.3	−2.3 ± 0.06
SPD_0420 (*pflB*)	Pyruvate formate lyase (PFL)	−2.9	0.0 ± 0.0

^a^Fold difference ≥2 were considered to be significant, ‘−’ sign indicates down regulation of genes, ‘±’ represents the standard deviation for three individual measurements.

**Table 3:** Expression levels (in Miller Units) of pneumococcal transcriptional *lacZ*-fusions to the promoters of transcriptional regulators grown anaerobically in CDM supplemented with 55 mM of glucose, galactose, mannose or *N*-acetyl glucosamine, or 10 mM sodium formate (SF)^a^.

Strains	CDM no sugar	Glucose	Galactose	Mannose	GlcNAc	Glucose + SF
pPP1::*lacZ*-wt	0.7 ± 0.08	0.4 ± 0.06	1.7 ± 0.04	0.8 ± 0.1	1.4 ± 0.2	0.5 ± 0.07
P*ccpA*::*lacZ*-wt	12.3 ± 1.3	99.4 ± 1.7	457.7 ± 9.4	125.5 ± 5.5	111.1 ± 4.5	241.4 ± 4.5
P*glnR*::*lacZ*-wt	17.3 ± 2.4	89.7 ± 4.4	559.5 ± 15.8	371.9 ± 10.7	118.9 ± 3.8	19.7 ± 0.8

^a^The activity is expressed as nmol *p*-nitrophenol/min/ml. Values are the average of at least three independent experiments, each with three replicates. ‘±’ indicates standard error of means (SEM). ‘wt’ indicates wild type D39 strain.

**Table 4:** Expression levels (in Miller Units) of pneumococcal transcriptional *lacZ*-fusions to the promoters of *ccpA* and *glnR* in different backgrounds grown anaerobically in CDM supplemented with 55 mM of glucose or galactose^a^.

Strains	Glucose	Galactose
pPP1::*lacZ*-wt	0.7 ± 0.05	0.9 ± 0.08
P*ccpA*::*lacZ*-wt	102.0 ± 1.5	451.2 ± 7.3
P*ccpA*::*lacZ*-Δ*ccpA*	40.2 ± 3.0	14.7 ± 2.2
P*ccpA*::*lacZ*-Δ*glnR*	9.9 ± 0.2	324.1 ± 10.2
P*glnR*::*lacZ*-wt	93.4 ± 1.8	565.0 ± 7.1
P*glnR*::*lacZ*-Δ*ccpA*	53.7 ± 2.5	147.8 ± 13.7
P*glnR*::*lacZ*-Δ*glnR*	89.7 ± 4.4	572.3 ± 4.2

^a^The activity is expressed as nmol *p*-nitrophenol/min/ml. Values are average of at least three independent experiments each with three replicates. ‘±’ indicates standard error of means (SEM).
