# Supplementary material for: Pneumococcal galactose catabolism is controlled by multiple regulators acting on pyruvate formate lyase
Source: Sci Rep. 2017 Feb 27;7:43587. doi: 10.1038/srep43587 (PMC5327383; doi:10.1038/srep43587)
Supplement: Supplementary Information [file srep43587-s1.pdf]

Supplementary Information

Pneumococcal galactose catabolism is controlled by multiple regulators acting on pyruvate formate lyase

Firas A.Y. Al-Bayati[1, 2], Hasan F. H. Kahya[1, 2], Andreas Damianou[1], Sulman Shafeeq[3], Oscar P. Kuipers[3], Peter W. Andrew[1], and Hasan Yesilkaya[1*]

[1]Department of Infection, Immunity & Inflammation, University of Leicester, Leicester, LE1 9HN, UK,

[2]Department of Biology, College of Education, University of Mosul, Iraq

[3]Molecular Genetics, University of Groningen, Nijenborgh 7, 9747 AG, Groningen, the Netherlands.

Supplementary Table S1. Fold change assessment of expression for selected genes in mutant pneumococcal strains grown anaerobically in CDM supplemented with 55 mM galactose relative to wild type strain D39 by quantitative real time reverse transcriptase PCR.

**Genes**		
	**Δ*ccpA***	**Δ*glnR***
pflB	-6.2* ± 0.4	3.6 ± 0.2
pflA	3.8 ± 0. 1	2.4 ± 0.15
ccpA	-5.9±0.3	-1.8± 0.1
glnR	-4.7± 0.2	1.2 ± 0.08

* '-' indicates down regulation of genes, ± represents the standard deviation for three individual measurements.

Supplementary Table S2. List of strains or plasmids used and constructed in this study.

Strains/Plasmids	Description/Use[a]	Source
S. pneumoniae		
D39	Serotype 2 strain	Laboratory stock
Δ*pflB*	D39; SPD0420:Spec^R	[1]
Δ*ccpA*	D39; SPD1797:Spec^R	This study
Δ*ccpA*K	D39; SPD1797:Kan^R	This study
Δ*plcR*	D39; SPD1745:Spec^R	This study
Δ*merR*	D39; SPD1637:Spec^R	This study
Δ*gntR*	D39; SPD1524:Spec^R	This study
Δ*glnR*	D39; SPD0447:Spec^R	This study
Δ*marR*	D39; SPD0379:Spec^R	This study
Δ*ccpA*Δ*glnR*	D39; SPD1797:Kan^R; SPD0447:Spec^R	This study
Δ*ccpA*Comp	D39; *ccpA* + Δ*ccpA*:Spec^R; Kan^R	This study
Δ*plcR*Comp	D39; *plcR* + Δ*plcR*:Spec^R; Kan^R	This study
Δ*gntR*Comp	D39; *gntR* + Δ*gntR*:Spec^R; Kan^R	This study
Δ*glnR*Comp	D39; *glnR* + Δ*glnR*:Spec^R; Kan^R	This study
pPP1::*lacZ*-wt	D39; Δ*bgaA*::pPP1-*lacZ*; Tet^R	This study
P*ccpA*::*lacZ*-wt	D39; Δ*bgaA*::P*ccpA-lacZ*; Tet^R	This study
P*plcR*::*lacZ*-wt	D39; Δ*bgaA*::P*plcR-lacZ*; Tet^R	This study
P*merR*::*lacZ*-wt	D39; Δ*bgaA*::P*merR-lacZ*; Tet^R	This study
P*gntR*::*lacZ*-wt	D39; Δ*bgaA*::P*gntR-lacZ*; Tet^R	This study
P*glnR*::*lacZ*-wt	D39; Δ*bgaA*::P*glnR-lacZ*; Tet^R	This study
P*marR*::*lacZ*-wt	D39; Δ*bgaA*::P*marR-lacZ*; Tet^R	This study
P*pflB*::*lacZ*-wt	D39; Δ*bgaA*::P*pflB-lacZ*; Tet^R	This study
P*pflA*::*lacZ*-wt	D39; Δ*bgaA*::P*pflA-lacZ*; Tet^R	This study
P*ccpA::lacZ*-Δ*ccpA*	Δ*ccpA*:Spec^R; Δ*bgaA*::P*ccpA-lacZ*; Tet^R	This study
P*ccpA::lacZ*-Δ*glnR*	Δ*glnR*:Spec^R; Δ*bgaA*::P*ccpA-lacZ*; Tet^R	This study
P*glnR::lacZ*-Δ*ccpA*	Δ*ccpA*:Spec^R; Δ*bgaA*::P*glnR-lacZ*; Tet^R	This study
P*glnR::lacZ*-Δ*glnR*	Δ*glnR*:Spec^R;Δ*bgaA*::P*glnR-lacZ*; Tet^R	This study
P*pflB::lacZ*-Δ*ccpA*	Δ*ccpA*:Spec^R; Δ*bgaA*::P*pflB-lacZ*; Tet^R	This study

Strains/Plasmids	Description/Use	Source
P*pflB::lacZ*-Δ*gntR*	Δ*gntR*:$Spec^R$; Δ*bgaA*::P*pflB-lacZ*; Tet^R	This study
P*pflB::lacZ*-Δ*glnR*	Δ*glnR*:$Spec^R$; Δ*bgaA*::P*pflB-lacZ*; Tet^R	This study
P*pflA::lacZ*-Δ*ccpA*	Δ*ccpA*:$Spec^R$; Δ*bgaA*::P*pflA-lacZ*; Tet^R	This study
P*pflA::lacZ*-Δ*glnR*	Δ*glnR*:$Spec^R$; Δ*bgaA*::P*pflA-lacZ*; Tet^R	This study
Escherichia coli		
One Shot® TOP10	Plasmid propagation	Invitrogen, UK
BL21 (DE3) pLysS	Protein expression	Agilent Tech, USA
Plasmids		
pDL278	Amplification of $Spec^R$ (*aadA*)	[2]
pLEICS-01	6His-Tag for protein expression; Amp^R	PROTEX, UK
pCEP	Genetic complementation; Kan^R	[3]
pPP1	Promoterless *lacZ* for transcriptional fusions; Amp^R Tet^R	[4]

[a] $Spec^R$: confers spectinomycin resistance gene (*aadA*), Kan^R: confers kanamycin resistance gene (*aph*), Amp^R and Tet^R: confers ampicillin and tetracycline resistance genes respectively.

Supplementary Table S3. List of primers used in this study.

Primer name	Sequence (5′–3′)
RT-PCR primers	
gyrB-RT-F	TGATGACCGATGCCGATG
gyrB-RT-R	TTGGGCAATATAAACATAACCAGC
SPD1797-RT-F	TATGATGTCGCTCGTGAAGC
SPD1797-RT-R	GCAACTGCATTTGGACGATA
SPD1745-RT-F	TTTTATTCCCTCCGCAGACTT
SPD1745-RT-R	TGTCATCTAATAGGCGAGCAGA
SPD1637-RT-F	GGTCTTGTGCCACCGATTAC
SPD1637-RT-R	TCGTTTCATCTCCCTTTTGG
SPD1594-RT-F	TTGAAGAGAGGGTTGGCAAG
SPD1594-RT-R	TTTTGCGCAATTTGACGATA
SPD1524-RT-F	AACAACTTCCAACCGTGAGG
SPD1524-RT-R	GGGCGATTAGCTCCTTATCC
SPD0447-RT-F	CATGGATCGTCTGCTTGAAA
SPD0447-RT-R	CCCTGTTGGAGGAGTTCATT
SPD0379-RT-F	TCATCGGTAAGGCTCCAGAT
SPD0379-RT-R	GTCAAATGCAGATGCACCAC
SPD0420-RT-F	TCCCTGCTGGATTTATCGAC
SPD0420-RT-R	TGGGTCTGGTTCGTATCCAT
SPD1774-RT-F	GGAAAACCTGTCTGGATTCG
SPD1774-RT-R	TTCACGCCACTTGAACTCAC
SPD1078-RT-F	ACGCGTTATCGGTTCAGGTA
SPD1078-RT-R	GTGACCAAACAGCGAACTCA
SPD1050-RT-F	GGTTCTGAGTGTGTGGCTGA
SPD1050-RT-R	AAAGCGTGGGTCTGAAAAGA
SPD1634-RT-F	GGGCTGATCAACGTGCTATT
SPD1634-RT-R	CTCAGCACGACGTTCATTGT
SPD1834-RT-F	TGCCAAAGCATCAGTAGCAG
SPD1834-RT-R	GTGTGGCGCATGTTGTTTAC

Supplementary Table S3 continued

Primer name	Sequence (5′–3′)
SPD1853-RT-F	GGATTTCGTGGTTTGGTTGT
SPD1853-RT-R	TTTCAAGCGTTCAACGTCAC
SPD0559-RT-F	GCTGAGAAAGTGGTGGTTGTT
SPD0559-RT-R	AGTCCATCAACTGAGCCAGAA
SOEing PCR primers	
Spec-F	ATCGATTTTCGTTCGTGAATACATGTTAT
Spec-R	GTTATGCAAGGGTTTATTGTTTTCTA
LF-SOE1797-F	GAATCGCCCGGGGCTTATCCAAC
LF-SOE1797-R	TATTCACGAACGAAAATCGATTACTGTATCATCTGCATTCATTC
RF-SOE1797-F	AACAATAAACCCTTGCATAACTCAACACGAAAACGTAAATAGAA
RF-SOE1797-R	TATGACAGATGAGCTGATTGATA
LF-SOE1745-F	GCGTCCAACGTGGCTCTGCACCA
LF-SOE1745-R	TATTCACGAACGAAAATCGATTTCTCTGCGAGTGTATTCATTA
RF-SOE1745-F	AACAATAAACCCTTGCATAACAAGGAACTAGATACTGTTTAGTT
RF-SOE1745-R	GCTAGGACACTATGGACTTCTTG
LF-SOE1637-F	CCACGTTTTTGGTCTACACTCAA
LF-SOE1637-R	TATTCACGAACGAAAATCGATACTGGCAGATTTAATATTCACAC
RF-SOE1637-F	AACAATAAACCCTTGCATAACTATAAGGAAGGAAAATTTTAAAT
RF-SOE1637-R	TGCTCCACGCATCTTAGCCGCGA
LF-SOE1594-F	ATCAGCAGATTTGTTTGAGTATA
LF-SOE1594-R	TATTCACGAACGAAAATCGATCAATACCCGACCATTAAACATTT
RF-SOE1594-F	AACAATAAACCCTTGCATAACTACAAAAGAAATAATTTATAATT
RF-SOE1594-R	CCCTTAGATTTGATTCCGATTCT
LF-SOE1524-F	ATTTGTTGGAGCAAGCTCTCTAA
LF-SOE1524-R	TATTCACGAACGAAAATCGATGTTGTCAAATGTCCAGGACATCT
RF-SOE1524-F	AACAATAAACCCTTGCATAACGATTATATTAAAGGAGTTTAAGC
RF-SOE1524-R	ATTGGCTCGATATCAGAAATCAA
LF-SOE0447-F	TCGATAAATCCCAGTTCAAACTT

Supplementary Table S3 continued

Primer name	Sequence (5′–3′)
LF-SOE0447-R	TATTCACGAACGAAAATCGATGCGAAATTCTTTTTCCTTCATTT
RF-SOE0447-F	AACAATAAACCCTTGCATAACTCACCTTTTGGTCGCGGTTAGGC
RF-SOE1524-R	ATTGGCTCGATATCAGAAATCAA
LF-SOE0447-F	TCGATAAATCCCAGTTCAAACTT
LF-SOE0447-R	TATTCACGAACGAAAATCGATGCGAAATTCTTTTTCCTTCATTT
RF-SOE0447-F	AACAATAAACCCTTGCATAACTCACCTTTTGGTCGCGGTTAGGC
RF-SOE0447-R	TCCCATTTTGGTCAAGACATTCA
LF-SOE0379-F	GTTTTAATGGAAGTTGACGGTGC
LF-SOE0379-R	TATTCACGAACGAAAATCGATATTGATTCGTTGGTAGTCCATTT
RF-SOE0379-F	AACAATAAACCCTTGCATAACTTTTTGGAGGATTTGAAATAATG
RF-SOE0379-R	AATGCCCATAAGTTAAACACTCG
Kan-F	GAGGTGCTACCATGGCGCGCA
Kan-R	CTAAAACAATTCATCCAGTAA
LF-SOE1797K-F	GAATCGCCCGGGGCTTATCCAAC
LF-SOE1797K-R	TGCGCGCCATGGTAGCACCTCTACTGTATCATCTGCATTCATTC
RF-SOE1797K-F	TTACTGGATGAATTGTTTTAGTCAACACGAAAACGTAAATAGAA
RF-SOE1797K-R	TATGACAGATGAGCTGATTGATA
Complementation primers	
SPD1797-Comp-F	CGGCATGCGAATAAGTAGAAATATGAAA
SPD1797-Comp-R	CGGGATCCCTATTTACGTTTTCGTGTTG
SPD1745-Comp-F	CGCCATGGGTAATTTTTAACTTTTTTTT
SPD1745-Comp-R	CGGGATCCCTAAACAGTATCTAGTTCCT
SPD1524-Comp-F	CGCCATGGTTCTTTTCATTATACCATTT
SPD1524-Comp-R	CGGGATCCTTAAACTCCTTTAATATAAT
SPD0447-Comp-F	CGCCATGGATATTGATCGTATTCGTCTC
SPD0447-Comp-R	CGGGATCCCTAACCGCGACCAAAAGGTG
Comp-Seq-F	GCTTGAAAAGGAGTATACTTAT
Comp-Seq-R	AGGAGACATTCCTTCCGTATC

Supplementary Table S3 continued

Primer name	Sequence (5′–3′)
Cloning into pLEICES-01 primers	
SPD1797-C-F	TACTTCCAATCCATGAATGCAGATGATACAGTAA
SPD1797-C-R	TATCCACCTTTACTGTCATTTACGTTTTCGTGTTGA
SPD1745-C-F	TACTTCCAATCCATGAATACACTCGCAGAGAAATT
SPD1745-C-R	TATCCACCTTTACTGTCAAACAGTATCTAGTTCCTT
SPD1637-C-F	TACTTCCAATCCATGAATATTAAATCTGCCAGTGA
SPD1637-C-R	TATCCACCTTTACTGTCAAAATTTTCCTTCCTTATA
SPD1594-C-F	TACTTCCAATCCATGTTTAATGGTCGGGTATTGAA
SPD1594-C-R	TATCCACCTTTACTGTCATAAATTATTTCTTTTGTACA
SPD1524-C-F	TACTTCCAATCCATGTCCTGGACATTTGACAACAA
SPD1524-C-R	TATCCACCTTTACTGTCAAACTCCTTTAATATAATCA
SPD0447-C-F	TACTTCCAATCCATGAAGGAAAAAGAATTTCGCCGA
SPD0447-C-R	TATCCACCTTTACTGTCAACCGCGACCAAAAGGTGA
SPD0379-C-F	TACTTCCAATCCATGGACTACCAACGAATCAATGA
SPD0379-C-R	TATCCACCTTTACTGTCATTTCAAATCCTCCAAAAATT
EMSA primers	
P*ccpA*-EMSA-F	TGAAATTTTTTCAATCAATCTTCA
P*ccpA*-EMSA-R	GTGAAAATTTCGTTTTCATATTT
P*plcR*-EMSA-F	CCACGTTTTTTTCTCATGCA
P*plcR*-EMSA-R	AAAATTACATAAAAATACTT
gyrB-EMSA-F	ATGACAGAAGAAATCAAAAATCTGC
gyrB-EMSA-R	CCTGGACGCATACGAACAG
P*merR*-EMSA-F	AAATTTTTCGATTAGATACA
P*merR*-EMSA-R	CATTCACTGAATTCACGCTA
P*gntR*-EMSA-F	CCCAAAACAATTCTTCTTTTT
P*gntR*-EMSA-R	GGACATCTTGGTCTCCT
P*glnR*-EMSA-F	CATTATCAATTGACGTTTGT
P*glnR*-EMSA-F	CCATATTTCGGCGAAATTCT

Supplementary Table S3 continued

Primer name	Sequence (5′–3′)
P*marR*-EMSA-F	AAAATCCTTGCATCATTCTT
P*marR*-EMSA-R	ATTTTTCATATCCCTCCTTCT
P*pflB*-EMSA-F	CAGTTTGAAATAAAATATAG
P*pflB*-EMSA-R	GACAAGTATACCATAAAGTA
P*pflB*(*cre1*-)-EMSA-F	AAAAGGACTTTATTTTTTTC
P*pflB*(*cre1*-)-EMSA-R	AAGTGCAAAATCCCTATTTT
P*pflA*-EMSA-F	GAAAAACAGATTGCTTTCTA
P*pflA*-EMSA-R	GTTACCTTCCTTGAAAACGT
P*pflA*(*cre1*-)-EMSA-F	TAATCTGATGATAGATTGAA
P*pflA*(*cre1*-)-EMSA-R	TTATTATATCACGACTTGAA
Transcriptional lacZ fusion primers	
P*ccpA*-Fusion-F	CGGCATGCCCTAGGTTTTCTATGAAATT
P*ccpA*-Fusion-R	CGGGATCCAAATGGTTACTGTATCATCT
P*plcR*-Fusion-F	CGGCATGCCTTTCTTTTTCCTTGTTTTT
P*plcR*-Fusion-R	CGGGATCCATCTGAATTTCTCTGCGAGT
P*merR*-Fusion-F	CGGCATGCGTTAATGATAAAAATTTTTC
P*merR*-Fusion-R	CGGGATCCACAAATCACTGGCAGATTTA
P*gntR*-Fusion-F	CGGCATGCCGCATCCTTTCCCTCCTTAT
P*gntR*-Fusion-R	CGGGATCCGTTTTTTGTTGTCAAATGTC
P*glnR*-Fusion-F	CGGCATGCCGTATTCGTCTCTTTTTAGA
P*glnR*-Fusion-R	CGGGATCCTATTTCGGCGAAATTCTTTT
P*marR*-Fusion-F	CGGCATGCGGAGTGCGAGCTCATTCGGA
P*marR*-Fusion-R	CGGGATCCAATATTCATTGATTCGTTGG
P*pflA*-Fusion-F	CGGCATGCTCTGATGATAGATTGAAAAT
P*pflA*-Fusion-R	CGGGATCCGTCCATAATCAATTGTTTCT
P*pflB*-Fusion-F	CGGCATGCGAAAAAAGGACTTTATTTTT
P*pflB*-Fusion-R	CGGGATCCGTGCTTCAACAACTGTCTTA
Fusion-Seq-F	CTACTTGGAGCCACTATCGA
Fusion-Seq-R	AGGCGATTAAGTTGGGTAAC

Supplementary Figure S1: SDS-PAGE (A) and Western blot (B) analysis of recombinant regulatory proteins. CcpA (38.8 kDa), PlcR (35.8 kDa), NmlR (15.2 kDa), GntR (15.7 kDa), GlnR (15.6 kDa) and MarR (18.4 kDa). SDS gel stained with Coomassie Brilliant Blue. Western hybridisation was done using Anti-His Tag monoclonal antibody (Sigma-Aldrich, UK). The blotted nitrocellulose membrane (Roche Applied Science, USA) was incubated for 2 h with the primary antibody. The membrane was washed with PBS-Tween20 (PBS 1000 ml, Tween 500 µl), and then incubated with the secondary antibody. The membrane was visualized with 5-Bromo-4-Chloro-Indolyl-Phosphatase (BCIP) (Sigma-Aldrich, UK).

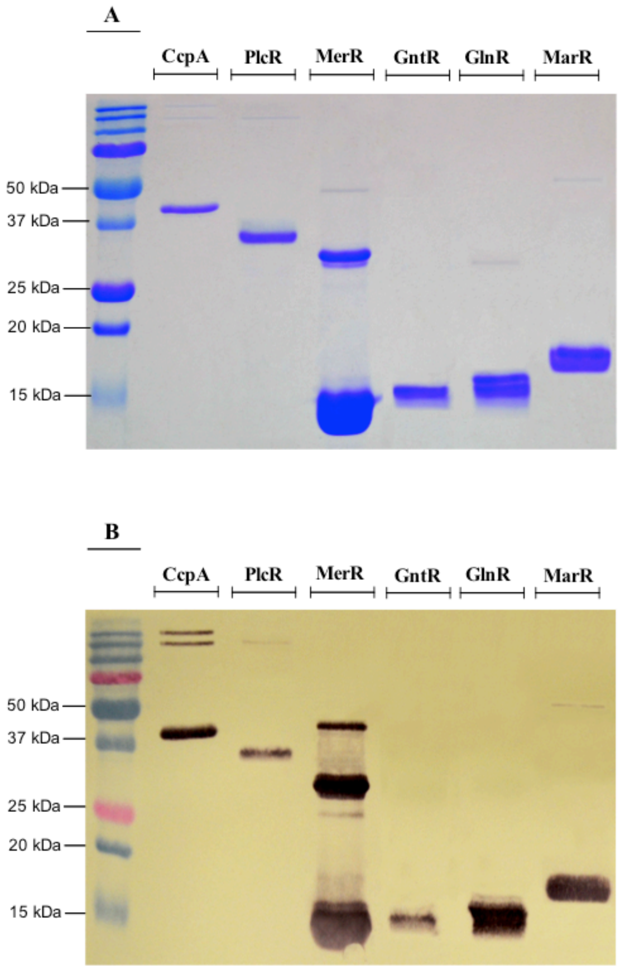

Supplementary Figure S2: EMSA analysis showing the direct interaction of GntR with P*pflB*. Each lane contains approximately 30 ng P*pflB*. GntR was used between 0.1 to 0.5 µM. The coding sequence of *gyrB* (30 ng) was used as a negative control. Gels were stained with SYBR Green EMSA for visualizing DNA.

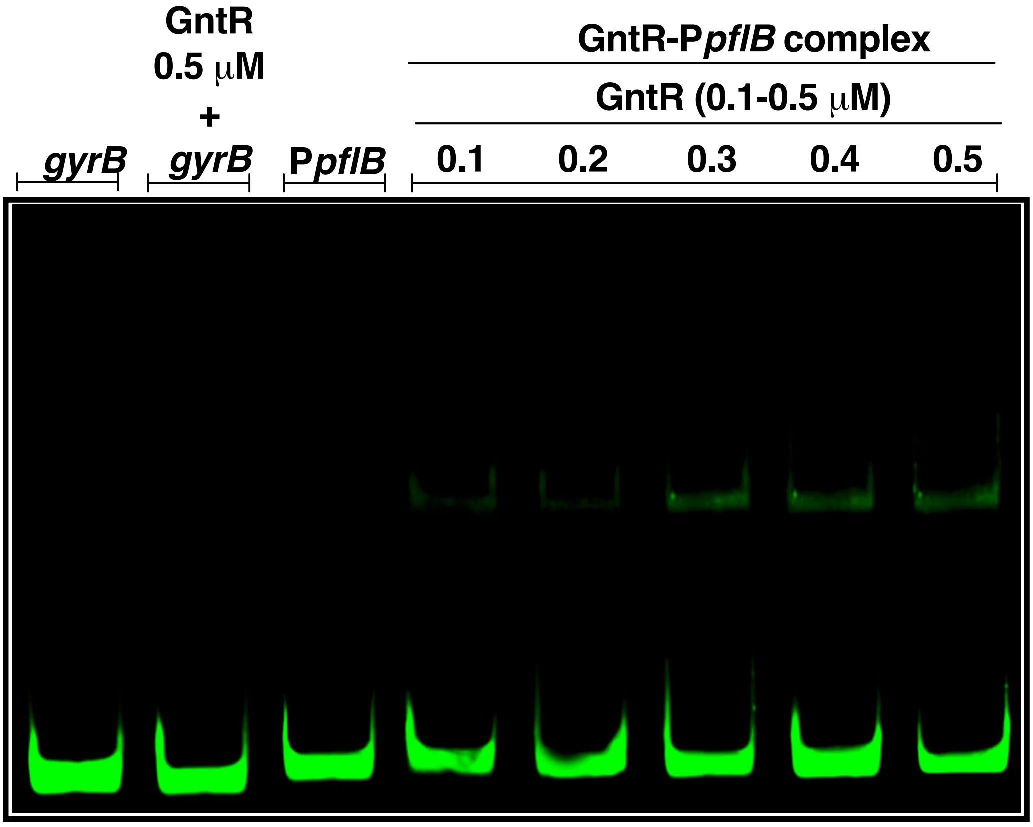

Supplementary Figure S3: The impact of sodium formate on binding affinity of CcpA (a) and GlnR (b) for P*pflB*. The presence of 10 mM sodium formate increased CcpA and decreased GlnR binding affinity for P*pflB*. This experiment was reproduced 3 times.

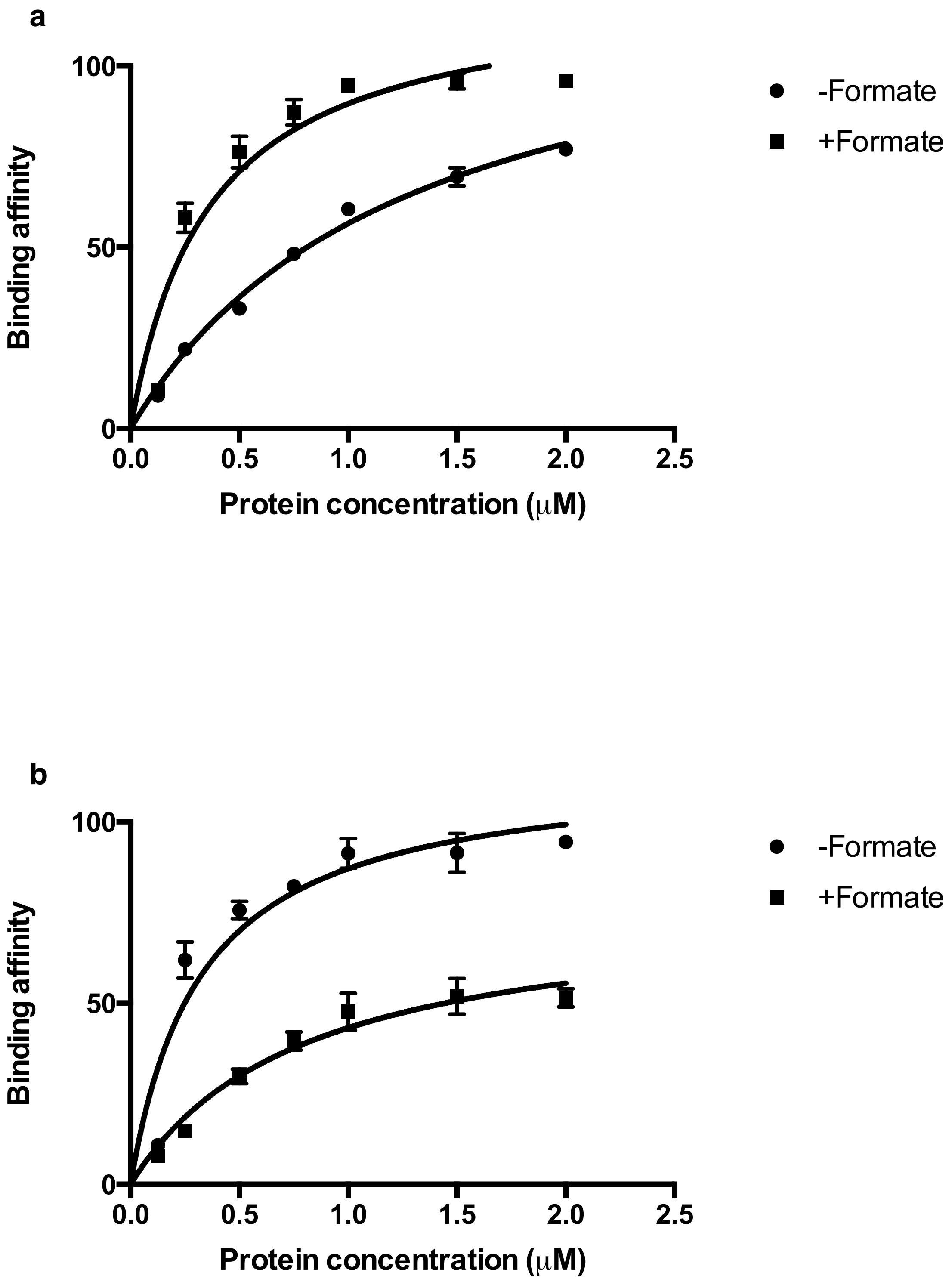

Supplementary Figure S4: Schematic representation showing the analysis of predicted promoter region and binding sites of P*pflB* (A) and P*pflA* (B) used in EMSA. The core promoter region containing the -10 and -35 elements is indicated. The putative *cre* sequences are indicated in red. The *cre1* sequence, was excluded from P*pflB*(*cre1*$^{-}$) and P*pflA*(*cre1*$^{-}$). F: indicates forward primer while R: refers to the reverse primer used for amplifying the promoter probe. T: potential terminator structure. The black arrow presents the direction of transcription.

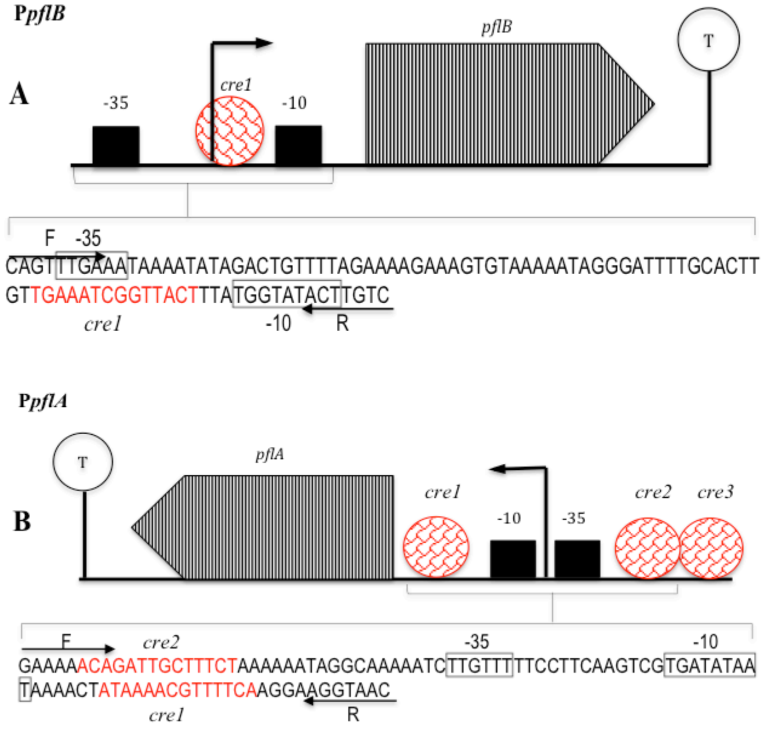

SUPPLEMENTARY REFERENCES

1. Yesilkaya, H. *et al.* Pyruvate formate lyase is required for pneumococcal fermentative metabolism and virulence. *Infect. Immun.* **77**, 5418–5427 (2009).
2. Yesilkaya, H. *Studies on the role of superoxide dismutase (SOD) in the virulence of* Streptococcus pneumoniae *and the effects of interferon gamma on sensitivity of phagocytes to the toxin pneumolysin. PhD Thesis. Department of Infection, Immunity and Inflammation,* (University of Leicester, 1999).
3. Guiral, S. *et al.* Construction and evaluation of a chromosomal expression platform (CEP) for ectopic, maltose-driven gene expression in *Streptococcus pneumoniae*. *Microbiology* **152**, 343–9 (2006).
4. Halfmann, A., Hakenbeck, R. & Brückner, R. A new integrative reporter plasmid for *Streptococcus pneumoniae*. *FEMS Microbiol. Lett.* **268**, 217–24 (2007).
